# A ribosomal gene panel predicting a novel synthetic lethality in non-BRCAness tumors

**DOI:** 10.1038/s41392-023-01401-y

**Published:** 2023-05-10

**Authors:** Chao Zhang, Qiang Guo, Lifeng Chen, Zheming Wu, Xiao-Jian Yan, Chengyang Zou, Qiuxue Zhang, Jiahong Tan, Tian Fang, Qunxian Rao, Yang Li, Shizhen Shen, Min Deng, Liewei Wang, Huanyao Gao, Jia Yu, Hu Li, Cheng Zhang, Somaira Nowsheen, Jake Kloeber, Fei Zhao, Ping Yin, Chunbo Teng, Zhongqiu Lin, Kun Song, Shuzhong Yao, Liangqing Yao, Lingying Wu, Yong Zhang, Xiaodong Cheng, Qinglei Gao, Jian Yuan, Zhenkun Lou, Jin-San Zhang

**Affiliations:** 1 Beijing Institute of Basic Medical Sciences, 100850 Beijing, China; 2 Department of Oncology, Mayo Clinic, Rochester, MN 55905 USA; 3 School of Pharmaceutical Sciences, Wenzhou Medical University, 325035 Wenzhou, Zhejiang China; 4 Key Laboratory of Endocrine Gland Diseases of Zhejiang Province, Zhejiang Provincial People’s Hospital, 310014 Hangzhou, Zhejiang China; 5 Department of Gynecology, Zhejiang Provincial People’s Hospital, 310014 Hangzhou, Zhejiang China; 6 Department of Gynecology, the First Affiliated Hospital of Wenzhou Medical University, 325000 Wenzhou, Zhejiang China; 7 Wuhan Kingwise Biotechnology Co., Ltd., 430206 Wuhan, Hubei China; 8 Cancer Biology Research Center (Key Laboratory of the Ministry of Education), Tongji Hospital, Tongji Medical College, Huazhong University of Science and Technology, 430030 Wuhan, Hubei China; 9 Department of Gynecological Oncology, Sun Yat-sen Memorial Hospital, Sun Yat-sen University, 510120 Guangzhou, Guangdong China; 10 Zhejiang Provincial Key Laboratory of Traditional Chinese Medicine for Reproductive Health Research, 310006 Hangzhou, Zhejiang China; 11 Zhejiang Provincial Key Laboratory of Precision Diagnosis and Therapy for Major Gynecological Diseases, 310006 Hangzhou, Zhejiang China; 12 Department of Molecular Pharmacology and Experimental Therapeutics, Mayo Clinic, Rochester, MN 55905 USA; 13 Department of Dermatology, University of California San Diego, San Diego, CA 92122 USA; 14 Key Laboratory of Saline-alkali Vegetation Ecology Restoration, Ministry of Education, College of Life Science, Northeast Forestry University, 150040 Harbin, China; 15 Division of Gynecology Oncology, Department of Obstetrics and Gynecology, Qilu Hospital, Shandong University, 250012 Jinan, Shandong China; 16 Department of Obstetrics and Gynecology, the First Affiliated Hospital, Sun Yat-Sen University, 510080 Guangzhou, Guangdong China; 17 Department of Gynecologic Oncology, Obstetrics and Gynecology Hospital of Fudan University, 200090 Shanghai, China; 18 Department of Gynecologic Oncology, National Cancer Center/National Clinical Research Center for Cancer/Cancer Hospital, Chinese Academy of Medical Sciences and Peking Union Medical College, 100021 Beijing, China; 19 Department of Radiation Oncology, Hubei Cancer Hospital, Tongji Medical College, Huazhong University of Science and Technology, 430030 Wuhan, Hubei China; 20 Department of Gynecologic Oncology, Women’s Hospital, School of Medicine, Zhejiang University, 310006 Hangzhou, Zhejiang China; 21 Key Laboratory of Arrhythmias of the Ministry of Education of China, Research Center for Translational Medicine, East Hospital, Tongji University School of Medicine, 200120 Shanghai, China; 22 Department of Biochemistry and Molecular Biology, Tongji University School of Medicine, 200120 Shanghai, China; 23 The Quzhou Affiliated Hospital of Wenzhou Medical University, Quzhou People’s Hospital, 324000 Quzhou, Zhejiang China; 24 Medical Research Center, and Key Laboratory of Interventional Pulmonology of Zhejiang Province, The First Affiliated Hospital of Wenzhou Medical University, 325000 Wenzhou, Zhejiang China

**Keywords:** Predictive markers, Target identification

## Abstract

Poly (ADP-ribose) polymerase (PARP) inhibitors are one of the most exciting classes of targeted therapy agents for cancers with homologous recombination (HR) deficiency. However, many patients without apparent HR defects also respond well to PARP inhibitors/cisplatin. The biomarker responsible for this mechanism remains unclear. Here, we identified a set of ribosomal genes that predict response to PARP inhibitors/cisplatin in HR-proficient patients. PARP inhibitor/cisplatin selectively eliminates cells with high expression of the eight genes in the identified panel via DNA damage (ATM) signaling-induced pro-apoptotic ribosomal stress, which along with ATM signaling-induced pro-survival HR repair constitutes a new model to balance the cell fate in response to DNA damage. Therefore, the combined examination of the gene panel along with HR status would allow for more precise predictions of clinical response to PARP inhibitor/cisplatin. The gene panel as an independent biomarker was validated by multiple published clinical datasets, as well as by an ovarian cancer organoids library we established. More importantly, its predictive value was further verified in a cohort of PARP inhibitor-treated ovarian cancer patients with both RNA-seq and WGS data. Furthermore, we identified several marketed drugs capable of upregulating the expression of the genes in the panel without causing HR deficiency in PARP inhibitor/cisplatin-resistant cell lines. These drugs enhance PARP inhibitor/cisplatin sensitivity in both intrinsically resistant organoids and cell lines with acquired resistance. Together, our study identifies a marker gene panel for HR-proficient patients and reveals a broader application of PARP inhibitor/cisplatin in cancer therapy.

## Introduction

Poly (ADP-ribose) polymerase (PARP) inhibitors are the first clinically approved cancer drugs designed to exploit synthetic lethality.^1^ The defects in homologous recombination (HR) repair sensitize cancer cells to PARP inhibitors. This is a classic example of the synthetic lethality paradigm first proposed by Bryant et al. and Farmer et al. in 2005.^2,3^ It has been previously demonstrated that BRCA-mutant tumor cells are 1000 times more sensitive to PARP inhibitors than BRCA-wild-type cells, which provided the impetus for the development of PARP inhibitors to be tested in clinical trials.^1^ Up until now, most clinical trials assessing PARP inhibitor synthetic lethality have focused on tumor types that exhibit significant germ-line BRCA1/2 mutations or significant fractions of other candidate BRCAness defects.^1^ Meanwhile, cisplatin, which has been used in the clinic for almost five decades, remains an important treatment regimen for at least 18 distinct tumor types.^4^ Both drugs are thought to best target tumor cells with homologous recombination (HR) defects. However, many patients without apparent HR defects also respond well to PARP inhibitor and cisplatin treatment.^5,6^ Thus, it is extremely important to develop strategies to precisely identify these patients who will benefit from such therapies, and to understand the mechanism by which these drugs kill cancer cells to design more effective therapy.

The traditional view is that the cancer cells were eliminated by PARP inhibitor mainly due to DNA repair failure. The classical synthetic lethality with PARP inhibitors is dependent on HR deficiency. In other words, cancer cells are preferentially eliminated by PARP inhibitor via fatal DNA double-strand damage due to HR repair deficiency, which is the main error-free DNA repair pathway responsible for the repair of double-stranded DNA damage. This paradigm of synthetic lethality was once considered to be equally applicable to all platinum drugs. However, recent studies found that the mechanisms behind different platinum drugs are diverse.^4,7^ For example, oxaliplatin, unlike cisplatin and carboplatin, has been shown to kill cancer cells through ribosomal biogenesis stress, but not DNA damage; this might explain distinct clinical implementation of oxaliplatin from cisplatin.^7^ Nevertheless, the mainstream view still believes that PARP inhibitors and cisplatin belong to the classic DNA damage agent. For PARP inhibitors, the current model is that PARP inhibitors “trap” PARP1 on DNA, preventing its autoPARylation and release from the site of damage. This causes a situation analogous to the mechanism of action of cancer drugs that inhibit Topoisomerase II. On the other hand, cisplatin prevents cell proliferation by reacting with DNA to produce Pt-DNA adducts to destroy DNA structure. However, there are many patients without apparent HR defects who are sensitive to PARP inhibitor/cisplatin. This has remained a longstanding urgent clinical question, and it suggests the presence of additional unknown mechanisms of action of PARP inhibitor/cisplatin.

Ribosome biogenesis is an important target of many anticancer drugs.^7^ The main function of the nucleolus is the rapid production of small and large ribosome subunits, a process that must be highly regulated to achieve proper cellular proliferation and growth. Efficient ribosome biogenesis consumes >60% of cellular energy (ATP) and thus it is tightly coupled with the energy status of a cell.^8^ The nucleolus also senses stress and is a central hub for coordinating stress responses.^9^ Cells tend to turn off this highly energy-consuming process in response to various stressors (e.g., DNA damage) thereby causing ribosomal stress,^9^ also known as the Impaired Ribosome Biogenesis Checkpoint (IRBC).^10^ Considering the general high proliferative characteristics of tumor cells, it is reasonable to postulate that IRBC might be a promising target for cancer therapy, especially for DNA damage agents widely used in cancer treatment. The DNA damage signaling-induced ribosomal stress might be one of the important factors that sensitize cancer cells to these kinds of drugs. However, to the best of our knowledge, predictive biomarkers for DNA damage signaling-induced ribosomal stress have not been reported. The identification of these biomarkers will extend the clinical application of these DNA damage agents.

Here, we identified a gene panel predicting response to PARP inhibitors/cisplatin treatment via a new mechanism of synthetic lethality. Cells with high expression of the factors in the gene panel are sensitive to PARP inhibitors/cisplatin therapy via DNA damage signaling-induced ribosomal stress despite their HR proficiency. Specifically, a PARP inhibitor or cisplatin selectively eliminates cells with high expression of genes in the panel via ATM-induced pro-apoptotic ribosomal stress, which along with pro-survival ATM-induced HR repair constitutes a new model to balance the cell fate in response to DNA damage. As a result, the gene panel is complementary to HR status, and their combined examination effectively predicts clinical response to PARP inhibitors and cisplatin. Furthermore, we identified several marketed drugs capable of upregulating the expression of the genes in the panel without causing HR deficiency in PARP inhibitor/cisplatin-resistant cell lines. These drugs enhance PARP inhibitor/cisplatin sensitivity in both intrinsically resistant organoids and cell lines with acquired resistance. This suggests that they induce the novel synthetic lethality represented by the gene panel we identified by causing ribosomal stress.

In conclusion, we identified a gene panel that allows the identification of additional non-BRCAness tumors similarly benefiting from such therapies via this new mechanism of synthetic lethality, which will extend the clinical application of PARP inhibitors/cisplatin to patients without apparent HR defects.

## Results

### Cisplatin and all PARP inhibitors share a common signature for drug sensitivity

**Fig. 1:**
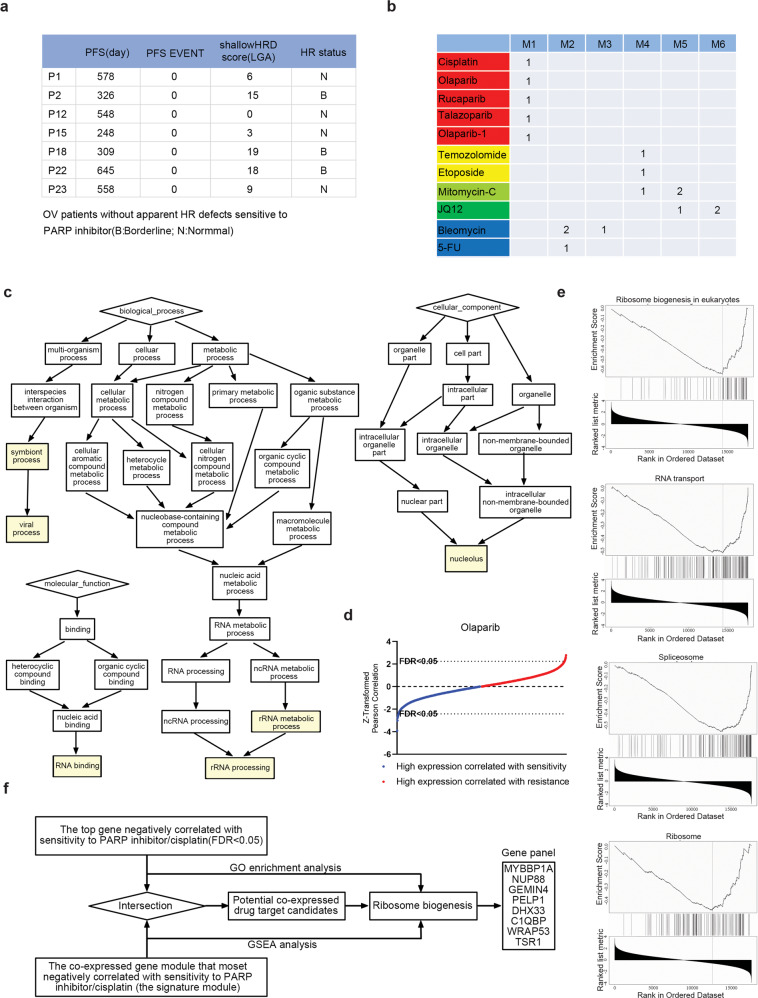
A gene panel was constructed based on the pathway analysis that identified ribosome biogenesis pathway as a potential predictor of cell response to PARP inhibitor/cisplatin. **a** A cohort of 50 ovarian cancer patients without apparent HR defects were enrolled as detailed in Materials and Methods. They are platinum-sensitive relapsed patients who were subsequently treated with PARP inhibitor maintenance monotherapy. **b** Classification for multiple DNA-damaging agents from GDBC according to their corresponding signature modules derived from WGCNA analysis. Among these datasets, there are two datasets of olaparib with drug ID 1017 and 1495, which were generated at two research centers (Massachusetts General Hospital and Wellcome Sanger Institute), respectively. We defined the olaparib with drug ID 1495 to be olaparib-1. The yellow-green for Mitomycin-C indicates transitional species between the drugs highlighted in yellow and green. M1~M6 represents 6 signature modules that are most negatively correlated with sensitivities to these drugs. **c** The identified biological processes, molecular functions, and cellular components GO terms of the signature module of PARP inhibitor/cisplatin derived from WGCNA analysis. **d** Distribution of Z-scaled olaparib sensitivity–gene-expression Pearson correlation values of all analyzed genes. **e** The top four negative enrichment pathways for olaparib identified by GSEA analysis on the drug sensitivity–gene-expression correlations. **f** The workflow to construct the gene panel predicting drug response to PARP inhibitor/cisplatin. We took the intersection of genes involved in their drug signature module and the top genes negatively correlated with sensitivity to these drugs (FDR < 0.05), from which eight genes involved in ribosome biogenesis were selected according to the results of both GSEA and GO enrichment analysis

Most clinical trials have focused on searching for or verifying biomarkers of BRCAness for PARP inhibitor response, however, it is well-known that many patients without apparent HR defects are also sensitive to platinum or PARP inhibitor. We thus enrolled a cohort of 50 ovarian cancer patients with platinum-sensitive, relapsed disease who achieved objective response (partial or complete response [PR or CR]) following the last platinum-based therapy prior to Olaparib Maintenance therapy from multiple hospitals in China mainland, and assessed their HR status via shallow HRD scores.^11^ According to multiple clinical studies on PARP inhibitor maintenance treatment for platinum-recurrent ovarian cancer patients, the median duration of progression-free survival of patients either with or without homologous recombination deficiency in the PARP inhibitor group ranged from 7.4 to 13.6 months, while that of patients in the placebo group are all less than 6 months.^12–15^ Interestingly, we found 7 patients without apparent HR defects but with progression-free survival durations exceeding 8 months, indicating that their tumors were likely sensitive to PARP inhibition (Fig. 1a). These data indicated that there is indeed a considerable proportion of patients without apparent HR defects who are sensitive to PARP inhibitor or platinum. Thus, it is important to develop a biomarker predicting the response of HR-proficient patients to PARP inhibitors, which would help greatly extend their clinical application.

To examine the diverse mechanisms of action of multiple DNA-damaging agents, we developed a computational pan-cancer analysis strategy to identify genes that correlate with drug sensitivities by analyzing RNA-seq data from the Cancer Cell Line Encyclopedia (CCLE)^16^ and drug sensitivity data (ln(IC_50_)) of 10 randomly chosen DNA-damaging agents with various putative targets (Supplementary Table S1) from the Genomics of Drug Sensitivity in Cancer (GDSC) dataset.^17^ Among these drug sensitivity data, there are two datasets of olaparib, which were generated at two research centers (Massachusetts General Hospital and Wellcome Sanger Institute), respectively. We found that the drug sensitivities of cell lines from these datasets formed a skewed distribution (Supplementary Fig. S1a–e, left panel), and parts of the variance-stabilizing transformation (VST)-transformed basal gene expression formed a bimodal distribution (Supplementary Fig. S1f, left panel), which can be attributed to the tissue of origin and histological subtype^18^ (Supplementary Fig. S2a–e, left panel). By using a linear regression model to remove these effects, we established a normalized distribution of both drug sensitivities (Supplementary Fig. S1a–e, right panel and Supplementary Fig. S2a–e, right panel) and basal gene expression (Supplementary Fig. S1f, right panel). We then performed weighted gene co-expression network analysis (WGCNA) to identify co-expressed gene modules that correlate with these drug sensitivity data (ln(IC_50_)) (Supplementary Fig. S3). The co-expressed gene module that was most negatively correlated with drug sensitivity data constituted a drug “signature”. Based on these drug “signatures”, the 11 DNA-damaging agents were classified into four distinct groups (Fig. 1b and Supplementary Table S2). Consistent with previous reports, multiple large and small ribosomal proteins were enriched in the intramodular gene hubs of 5-fluorouracil (5-FU) (Supplementary Table S2), which breaks the function of the ribosome directly.^19^ Interestingly, we found that cisplatin and all three PARP inhibitors (olaparib, rucaparib, and talazoparib) examined were classified into the same group (red group) (Fig. 1b and Supplementary Table S2), indicating that these drugs might function through a shared mechanism. We confirmed this by analyzing multiple human GEO transcriptome datasets (Supplementary Fig. S4). We found that more than 84% of the genes in this gene module (M1) are also co-expressed in the human gene co-expression network established by Coexpedia,^20^ indicating that the gene co-expression network obtained from cell lines is highly consistent with that established from patient datasets in vivo. Together, these results suggest PARP inhibitors and cisplatin might share a common signature for drug sensitivity.

### Ribosome biogenesis is highly associated with cellular sensitivity to PARP inhibitor/cisplatin

To define the biological pathways predictive of PARP inhibitor and cisplatin sensitivity, we performed GO enrichment analysis for the genes in the signature module of these drugs derived by WGCNA. The identified biological processes, molecular functions, and cellular components GO terms included rRNA processing (*P* = 4.48E-4), RNA-binding (*P* = 2.76E-5) and nucleolus (*P* = 6.54E-4) (Fig. 1c), indicating that nucleolar genes involved in ribosome biogenesis are enriched in the drug signature module of PARP inhibitors and cisplatin. We further correlated gene-expression levels with sensitivity data for PARP inhibitors and cisplatin by Pearson’s correlation analysis (Fig. 1d and Supplementary Table S3). The top negative enrichment pathways of these drugs were identified by using GSEA analysis on the drug sensitivity–gene-expression correlations and are highly consistent throughout, indicating that they are all RNA metabolism-related pathways (Fig. 1e and Supplementary Figs. S5–9). Our results suggest that ribosome biogenesis is a potential pathway to predict the drug response of cells to PARP inhibitor/cisplatin.

### Prediction of cellular response to cisplatin and PARP inhibitors in HR-proficient cancer via a gene panel

We developed a simple strategy to design a gene panel predicting drug response to PARP inhibitors/cisplatin. Specifically, it has been demonstrated that the systematic correlation of sensitivity data with basal gene-expression data is an efficient way to identify potential biomarkers of drugs.^18,21^ Specifically, there is a dependency of sensitive cell lines on the genes whose high expression negatively correlated with drug sensitivity data (i.e., ln(IC_50_)).^21^ Therefore, it is reasonable to postulate that the drug signature module most negatively correlated with drug sensitivity data might also include potential biomarkers of the drugs. We thus took the intersection of the genes involved in their drug signature module and the top genes negatively correlated with sensitivity to these drugs (FDR < 0.05). Ultimately, we obtained 21 co-expressed drug target candidates (Supplementary Table S4), of which 8 genes (MYBBP1A, NUP88, GEMIN4, PELP1, DHX33, C1QBP, WRAP53 and TSR1) have been specifically reported to be involved in ribosome biogenesis.^22–29^ Our prior analysis suggested that the ribosome biogenesis pathway is a potential predictor of response to PARP inhibitor/cisplatin. Therefore, we constructed a gene panel using these eight genes to predict cellular response to PARP inhibitor/cisplatin (Fig. 1f).

Based on our analysis, genes listed higher in our panel should be associated with increased PARP inhibitor/cisplatin sensitivity. We utilized Principal component analysis (PCA) scores to quantify the overall rank of this co-expressed gene panel.^6^ Across all the drugs and tumor types examined, the median ln(IC_50_) in cell lines with high expressions of genes in the panel was lower in the majority of cases (57 out of 73; Supplementary Figs. S10–13), consistent with our hypothesis that high expression of factors in the gene panel is correlated with hypersensitivity to PARP inhibitors/cisplatin. For ovarian cancer cell lines (Supplementary Table S5), the ln(IC_50_) values were not significantly lower in the high-expression group when compared to the low-expression group except for rucaparib (Supplementary Fig. S14a, upper panel). This might be partially caused by existing HR defects in some of these cell lines which confounded the results. We removed these confounders by screening out HR-proficient cell lines according to their levels of COSMIC mutation signature 3 (HR mutation signature). After this, the ln(IC_50_) values for talazoparib, rucaparib, and cisplatin were all significantly lower for the high-expression group when compared to the low-expression group (Supplementary Fig. S14a, lower panel). For breast cancer cell lines (Supplementary Table S6), the ln(IC_50_) values for all the drugs were significantly lower in cell lines with high expression of factors in the gene panel except for olaparib (Supplementary Fig. S14b, upper panel). After screening out HR-proficient breast cancer cell lines, the gene panel accurately predicted the cellular sensitivity of these cell lines to PARP inhibitor/cisplatin (Supplementary Fig. S14b, lower panel).

These results suggest that high expression of the genes in the panel correlates with cellular susceptibility to PARP inhibitor/cisplatin in HR-proficient cells and imply that the combined examination of HR status and the gene panel might precisely predict the response of patients to these drugs.

### HR-proficient cells with high expression of the assembled gene panel undergo cell death via ribosomal stress after PARP inhibitor/cisplatin treatment

As mentioned above, we developed a potential gene panel predicting drug response of PARP inhibitors/cisplatin based on the hypothesis that HR-proficient cells with high expression of the genes in the panel might undergo cell death in response to PARP inhibitor/cisplatin treatment via ribosomal stress. In order to test this hypothesis, we chose several ovarian, breast and colon cancer cell lines without apparent HR defects. The ovarian cancer cell lines include: OV90, OVKATE, OV56, A2780, and IGROV1; the colon cancer cell lines include: COLO678, HT-29 and HCT116; and the breast cancer cell lines include: ZR-7530, HCC1954 and BT549. Gene expression for our gene panel was assessed using qRT-PCR, and cell lines were separated into high- and low-expression groups for each cancer type according to the result (Supplementary Fig. S15a). We further verified that high expression of genes in the panel predicts susceptibility to PARP inhibitor/cisplatin in HR-proficient cells (Supplementary Fig. S15b).

**Fig. 2:**
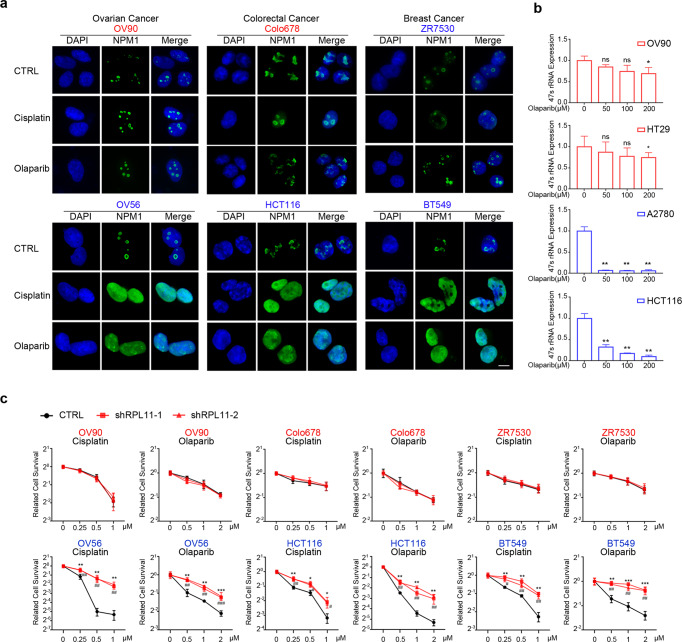
Cells showing high expression of genes in the panel were killed via DNA damage-induced ribosome biogenesis stress. **a** Indicated cells were treated with 50 μM of olaparib or cisplatin for 6 h (scale bar: 5 μm). The NPM1 localization was detected by immunofluorescence, and their nucleoli-restricted or diffused nuclear localization reflected the ribosomal stress level. The three cell lines shown in red were lowly expressed for the gene panel, whereas those in blue were the ones showing high expression for the gene panel. **b** Cells were treated with indicated concentrations of olaparib for 6 h, and the levels of 47 S pre-rRNA were determined by qRT-PCR to assess the extent of ribosomal stress. **c** The expression of RPL11 was knocked down with lentivirus-mediated shRNA expression to block the lethal effects of ribosomal stress in indicated cells, followed by colony-formation assay to determine their drug sensitivity to cisplatin or olaparib. *P < 0.05, **P < 0.01, ***P < 0.001, ^#^*P* < 0.05, ^##^*P* < 0.01, ^###^*P* < 0.001, ns not significant (compared with CTRL)

Ribosomal stress can be assessed by the loss of nucleolar integrity, which leads to the translocation of nucleophosmin (NPM) from the nucleolus into the nucleoplasm. We assessed the effect of these drugs on the nucleolar integrity in cell lines of both high- and low-expression groups. Immunofluorescence staining showed that PARP inhibitor/cisplatin treatment caused remarkable translocation of NPM from the nucleolus into the nucleoplasm in cell lines of the high-expression group, but this did not occur in cell lines of the low-expression group (Fig. 2a). Ribosomal stress was also evaluated by measuring the level of 47 S pre-rRNA which correlates with rRNA transcription rate. The qRT-PCR results showed that PARP inhibition significantly decreased the 47 S pre-rRNA levels in cell lines of high-expression group but not in cell lines of low-expression group (Fig. 2b). These results indicate that olaparib/cisplatin causes more severe ribosomal stress in cell lines with higher expression of genes in the panel.

Ribosomal stress results in the release of subunits of RPL11 (*RPL11*) that induces apoptosis.^4,30^ We thus examined the role of RPL11 (*RPL11*) in drug-induced cell death in cells of both high- and low-expression groups. RPL11 knockdown using shRNA conferred resistance to PARP inhibitor/cisplatin in cells of high but not low-expression group (Fig. 2c). Notably, RPL11 knockdown did not have any apparent effects on HR function in cells of the high-expression group (Supplementary Fig. S16a–c). These results support the hypothesis that the ribosomal stress pathway is a central mediator of PARP inhibitor/cisplatin-induced cytotoxicity in HR-proficient cells with high expression of genes in the panel. In summary, these results support that HR-proficient cells with high expression of genes in the panel undergo cell death via ribosomal stress when exposed to PARP inhibitor/cisplatin.

### ATM signaling balances cell fate by controlling both HR repair and ribosomal stress simultaneously during PARP inhibitor/cisplatin treatment

**Fig. 3:**
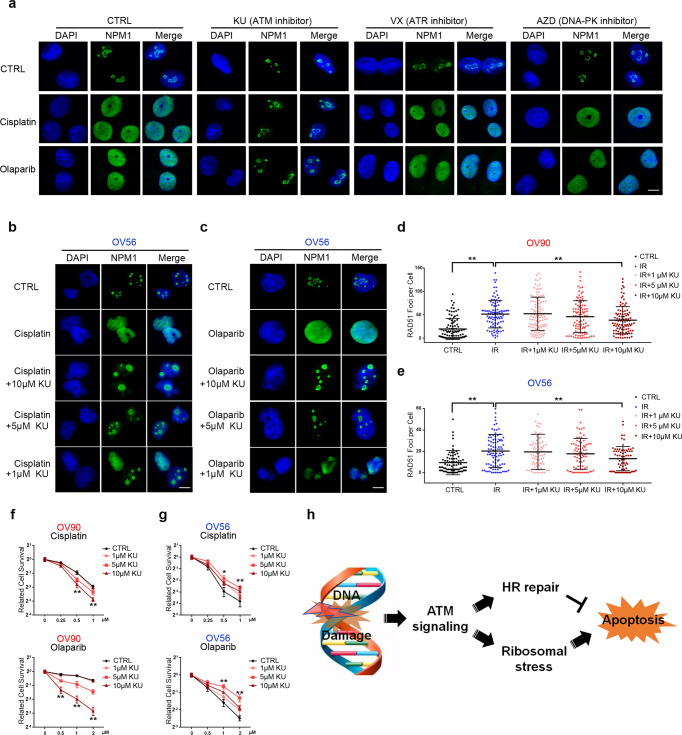
ATM signaling balances the cell fate by simultaneously controlling both HR repair and ribosomal stress during PARP inhibitor/cisplatin treatment. **a** OV56 cells were pretreated with KU55933 (ATM inhibitor), VX970 (ATR inhibitor), or AZD7648 (DNA-PK inhibitor) for 1 h (scale bar: 5 μm). The localization of NPM1 was detected by immunofluorescence to indicate the ribosomal stress induced by cisplatin or olaparib. **b**, **c** OV56 cells were pretreated with indicated concentrations of KU55933 for 1 h (scale bar: 5 μm). The localization of NPM1 was detected to indicate the ribosomal stress level caused by cisplatin or olaparib. **d**, **e** OV90 cells (**d**) and OV56 cells (**e**) were pretreated with indicated concentrations of KU55933 for 1 h followed by ionizing radiation (2 Gy) to trigger DNA double-strand breaks. RAD51 foci were stained and quantified to determine the HR status. **f**, **g** OV90 cells (**f**) and OV56 cells (**g**) were pretreated with indicated concentrations of KU55933, colony-formation assay was performed to determine the drug sensitivity to cisplatin or olaparib. **h** Proposed model of cell fate in response to PARP inhibitor/cisplatin treatment. Red labels: cell lines with low expression of the gene panel; Blue labels: cell lines with high expression of the gene panel. *P < 0.05, **P < 0.01, (com*p*ared with CTRL)

Although our analysis showed that ribosome biogenesis is highly associated with cellular sensitivity to PARP inhibitors/cisplatin in HR-proficient cells, their signature module is different from that of 5-FU, suggesting that they didn’t impair the function of ribosomes directly. PARP inhibitors and cisplatin are well-known DNA-damaging agents, causing accumulation of DNA single- and double-strand breaks. Therefore, we hypothesized that HR-proficient cells with high expression of genes in the panel might undergo cell death in response to PARP inhibitor/cisplatin treatment via DNA damage signaling-induced ribosomal stress. We thus checked whether the ribosomal stress caused by PARP inhibitors/cisplatin is dependent on DNA damage signaling. Immunofluorescence staining indicated that olaparib/cisplatin-induced γH2AX foci after 4 h of drug treatment in cell lines with either high or low expression of genes in the panel (Supplementary Fig. S17a). In addition, the ATM inhibitor (KU55933), but not ATR or DNA-PK inhibitor, effectively blocked the release of NPM upon PARP inhibitor/cisplatin treatment in the cell line with high expression of genes in the panel (Fig. 3a and Supplementary Fig. S17b). These results suggest that PARP inhibitor/cisplatin induces ribosomal stress in HR-proficient cell lines with high levels of the gene panel due to ATM signaling.

Prior studies have attempted to study potential synthetic lethality of ATM alterations with PARP inhibitor/cisplatin response based on the expectations that cells lacking ATM should exhibit a severe HR repair defect.^31^ However, many conflicting results have raised doubts about the role of ATM in HR repair.^32–37^ Our study indicates that ATM might exert two opposite effects on cell survival during PARP inhibitor/cisplatin treatment: one is ATM-dependent HR repair promoting cell survival, and the other one is ATM-dependent ribosomal stress inducing cell apoptosis (Fig. 3h). We thus utilized an ATM inhibitor (KU55933) to test the role of ATM signaling during PARP inhibitor/cisplatin treatment. Low dose of ATM inhibitor (1 µM) caused no significant changes on ribosomal stress (Fig. 3b, c) and cellular HR repair (Fig. 3d, e) induced by PARP inhibitor/cisplatin in cells with high expression of genes in the panel. Low-dose ATM inhibition also had no effect on cell survival with these drugs (Fig. 3f, g). Interestingly, our results showed that a moderate dose of ATM inhibitor (5 µM) caused no apparent effects on cellular HR repair (Fig. 3d, e), but effectively inhibited the ribosomal stress induced by PARP inhibitor/cisplatin in cell line with high expression of genes in the panel (Fig. 3b, c). This moderate dose of ATM inhibitor surprisingly conferred resistance to PARP inhibitor/cisplatin in cell lines with high expression of genes in the panel (Fig. 3g). Furthermore, a high dose of ATM inhibitor (10 µM) both blocked ribosomal stress and significantly compromised cellular HR repair (Fig. 3b, c, e), causing more cell death than the moderate dose of ATM inhibitor (5 µM) in cells with high expression of genes in the panel (Fig. 3g). This implies that cell death was caused by the defects in HR repair, although it still conferred mild resistance to PARP inhibitor/cisplatin in these cells possibly due to the inhibition of ribosomal stress (Fig. 3g). Conversely, a high level of ATM inhibitor (10 µM) significantly compromised the HR repair in cell lines with low expression of genes in the panel (Fig. 3d), remarkably sensitizing these cells to PARP inhibitor/cisplatin (Fig. 3f). On the other hand, all the three doses of ATM inhibitor caused no significant alterations on nucleolus status in cell line with low expression of genes in the panel after PARP inhibitor/cisplatin treatment (Supplementary Fig. S17c, d).

Therefore, these results further suggest that our gene panel is an important factor affecting cell survival in addition to HR status during PARP inhibitor/cisplatin treatment and that ATM signaling balances the cell fate by controlling both HR repair and ribosomal stress simultaneously (Fig. 3h). The double-edged sword effect of ATM signaling also provides a possible explanation for the conflicting results in multiple clinical studies regarding classical synthetic lethality of ATM mutation/inhibition with PARP inhibitor/cisplatin.^32–37^

### The combination of the gene panel and HR status effectively predicts clinical response to cisplatin

So far, our data suggest that the gene panel we identified is an additional important factor affecting cell survival besides HR status, thus the combination analysis of both might precisely predict sensitivity to PARP inhibitor/cisplatin. Specifically, the gene panel can be utilized to select responders from patients that do not traditionally fall into the known PARP inhibitor/cisplatin responder group based on HR defects. An alternative way is to first screen out all patients with high expression of genes in the panel as potential responders, and then HR status can be utilized to further select out responders from the remaining patients with low expression of genes in the panel (Supplementary Fig. S18).

**Fig. 4:**
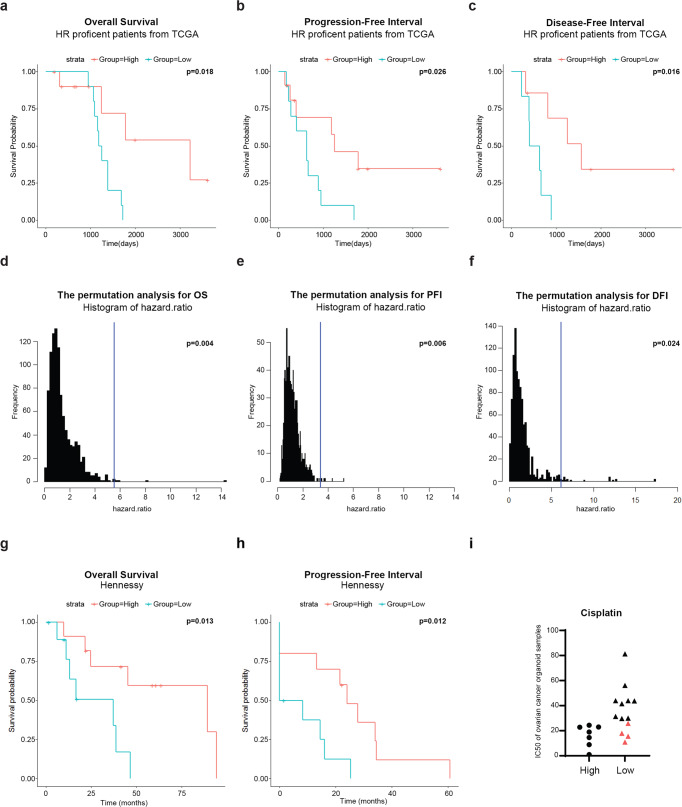
The combined examination of gene panel and HR status effectively predicts clinical drug response of cisplatin. **a** The overall survival analysis via univariate Cox regression with the gene panel for cisplatin-treated HR-proficient patients from TCGA (hazard ratio = 5.54; 95% CI 1.16–26.43; *P* = 0.018; *n* = 21). **b** The progression-free interval analysis using the gene panel for cisplatin-treated HR-proficient patients from TCGA (hazard ratio = 3.412; 95% CI 1.104–10.55; *P* = 0.026; *n* = 21). Log-rank *P* value is displayed. **c** The disease-free interval analysis with the gene panel for cisplatin-treated HR-proficient patients from TCGA (hazard ratio = 6.143; 95% CI 1.176–32.08; *P* = 0.016; *n* = 13). Log-rank *P* value is displayed. **d**–**f** The permutation test (1000 times) to compare the performance of randomly selected gene lists with our gene panel to predict the overall survival (*P* = 0.004), progression-free interval (*P* = 0.006) and disease-free interval (*P* = 0.024) of cisplatin-treated patients with normal HR function. One-tailed test. **g**, **h** The overall survival (hazard ratio = 4.39; 95% CI 1.238–15.56; *P* = 0.013; *n* = 21) and progression-free survival analyses (hazard ratio = 3.815; 95% CI 1.216–11.97; *P* = 0.012; *n* = 20) via univariate Cox regression with the gene panel for cisplatin-treated patients from Hennessy cohort suggested that patients with high expression of the gene panel are responders to cisplatin. **i** The combined examination predicts the response of ovarian cancer organoid samples to cisplatin. The red color indicates samples with known HR defects

We first examined the effect of combined examination of HR status and the gene panel on predicting patients’ response to cisplatin. We performed overall survival (OS) analysis of cisplatin-treated ovarian cancer patients from TCGA. Patients treated with other types of platinum agents were removed to limit confounding effects driven by heterogeneity of platinum agents.^4^ HR status was determined by the COSMIC mutation signature 3.^38^ As expected, patients can be roughly classified into four groups: only HR-proficient patients with low expression of genes in the panel are resistant to cisplatin, while patients from the other three groups are potential responders (Supplementary Fig. S19a). HR status can be utilized to predict the response of patients with low expression of genes in the panel (hazard ratio = 4.082; 95% CI 1.529–10.9; *P* = 0.0026, *n* = 42) (Supplementary Fig. S19b). Most importantly, the gene panel can be utilized to predict the response of HR-proficient patients (hazard ratio = 5.54; 95% CI 1.16–26.43; *P* = 0.018, *n* = 21) (Fig. 4a). We further performed an analysis using two surrogate endpoints of OS: progression-free interval (PFI) and disease-free interval (DFI) on these patients. Similarly, HR status is the preferred indicator for patients with low expression of factors in the panel (PFI: hazard ratio = 2.031, 95% CI 0.9434–4.373, *P* = 0.065, *n* = 42; DFI: hazard ratio = 6.551, 95% CI 1.813–23.67, *P* = 0.001, *n* = 24) (Supplementary Fig. S19c, d), while our gene panel accurately predicted the response of HR-proficient patients (PFI: hazard ratio = 3.412, 95% CI 1.104–10.55, *P* = 0.026, *n* = 21; DFI: hazard ratio = 6.143, 95% CI 1.176–32.08, *P* = 0.016, *n* = 13) (Fig. 4b, c). Permutation tests were performed to compare the performance (hazard ratio) of randomly selected lists of eight genes from the whole-coding genes with our gene panel to predict the drug response of patients with normal HR function (*P* = 0.004 for OS; *P* = 0.006 for PFI; *P* = 0.024 for DFI) (Fig. 4d–f). We further performed the permutation tests based on the top genes negatively correlated with sensitivity to PARP inhibitor/cisplatin, which puts forward more stringent requirements on the performance of the gene panel (*P* = 0.024 for OS; *P* = 0.021 for PFI; *P* = 0.045 for DFI) (Supplementary Fig. 19e–g). The permutation analysis indicated that the expressions of genes in the panel groups indeed differed in their response to cisplatin in HR-proficient patients.

Most previously published datasets of platinum-treated patients do not provide information on the types of platinum used by patients, except for the TCGA datasets analyzed above. Fortunately, we identified one cohort of platinum-treated ovarian cancer patients from Hennessy with microarray data,^39^ and we obtained information on the types of platinum of this cohort. Only cisplatin-treated patients were used for the following analysis. Although the HR status of these patients is unknown, patients with high expression of genes in the panel were still significantly more sensitive than those with low expression (OS: hazard ratio = 4.391, 95% CI 1.238–15.56, *P* = 0.013, *n* = 21; PFS: hazard ratio = 3.815, 95% CI 1.216–11.97, *P* = 0.012, *n* = 20) (Fig. 4g, h). These results support that the patients with high expression of genes in the panel are responders to cisplatin. Furthermore, we established an ovarian cancer organoid library treated with cisplatin. The results verify again that high expression of genes in the panel predict sensitivity to this drug, and that all the resistant samples have relatively low expression of genes in the panel (Fig. 4i and Supplementary Table S7). In addition, gene mutations involved in HR repair are found in all the four sensitive samples with low expression of genes in the panel (Fig. 4i and Supplementary Tables S7 and 8), except for O18. However, in O18 sample, we found a *SMAD3* mutation. *SMAD3* is not a classical HR gene but was recently reported to induce resistance to PARP inhibitors via promoting DNA double-strand break repair,^40^ possibly implying the BRCAness phenotype of O18. These results indicate that the combined examination of HR status and gene panel effectively screens out patients who are responders to cisplatin.

### The combination of the gene panel and HR status effectively predicts clinical response to PARP inhibitors

**Fig. 5:**
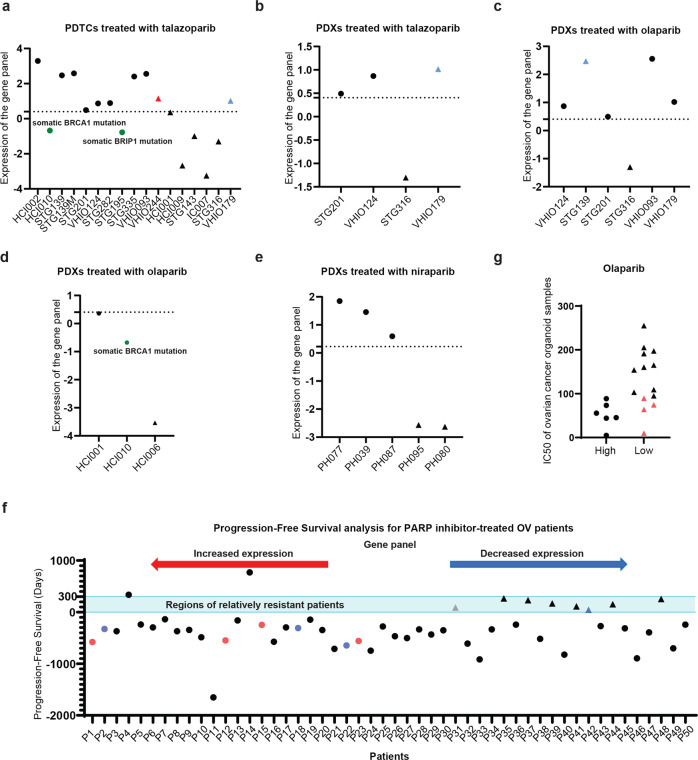
The combined examination of gene panel and HR status effectively predicts clinical drug response of PARP inhibitor. **a** The combined examination predicts the ex vivo response to PARP inhibitors in BRCA PDTX-derived tumor cells (PDTC) from BcaPE. **b**–**d** The combined examination predicts the in vivo response to PARP inhibitors in breast PDX models from BcaPE in three independent validation. **e** The combined examination predicts the in vivo response to PARP inhibitor in ovarian PDX models. **a**–**e** The values above the dot line represent predicted PDTX-derived tumor cells (PDTCs) or PDX models responsive to PARP inhibitor, while the values below the dot line represent predicted PDTCs or PDX models resistant to PARP inhibitor. Circular: sensitive PDTCs or PDX models; Triangle: resistant PDTCs or PDX models. The red color indicates misclassified PDTCs, blue color represents PDTCs or PDX models with drug-specific mechanism of resistance, and green color indicates the classification for PDTCs or PDX models that is corrected by HR status. **f** The combined examination predicts the real-world clinical outcomes in patients with platinum-sensitive relapsed ovarian cancer treated with PARP inhibitor maintenance monotherapy. **f** Circular: sensitive patients; Triangle: resistant patients. All patients were ranked from high to low according to their gene panel expression levels. The *y* axis values above the dot line (*y* = 0) represent the duration of progression-free survival of patients relapsed or died, while those below the dot line (*y* = 0) represent the duration of progression-free survival of patients without PFS events. The red color indicates patients profiled as HR proficiency, black color represents patients profiled as HR deficiency, gray color represents patients with unknown HR status, and blue color indicates patients with borderline HR function. **g** The combined examination predicts the response of ovarian cancer organoid samples to olaparib. The red color indicates samples with known HR defects

Next, we further examined the effect of our model on predicting the patients’ response to PARP inhibitors. We first validated it by predicting both ex vivo and in vivo responses of a panel of Patient-Derived Tumor Xenograft (PDTX)-derived tumor cells (PDTCs) of breast cancer from the BCaPE database to PARP inhibitors (talazoparib/olaparib).^41^ Specifically, we selected all PDTCs with either relatively high expression of genes in the panel or HR defects as PARP-sensitive PDTCs. This method successfully identified all the talazoparib-sensitive PDTCs ex vivo, among which HCI010 and STG195 had relatively low expression of genes in the panel and were predicted to be sensitive models due to their known somatic BRCA1 mutation and BRIP1 mutation, respectively (Fig. 5a and Supplementary Table S9). Our method only misclassified a single resistant model (VHIO244) (Fig. 5a and Supplementary Table S9). Furthermore, the in vivo responses of four PDTCs and six PDTCs from the BCaPE database was verified in PDX mouse models with talazoparib or olaparib, respectively (Fig. 5b, c and Supplementary Table S9).^41^ The in vivo response of HCI006, HCI001, and HCI010 from BCaPE database has also been determined in PDX mouse models in an independent study on olaparib (Fig. 5d and Supplementary Table S9).^39^ Although our method caused two misclassified resistant models (VHI0179 and STG139) in the three independent validations of in vivo response as well as the prior validation of ex vivo response, these errors might be caused by the drug-specific mechanism of resistance (Fig. 5a–c). Specifically, VHIO179, which was predicted to be sensitive to PARP inhibitor (Supplementary Table S9), was sensitive to olaparib (Fig. 5c), but resistant to talazoparib (Fig. 5a, b); STG139, which was predicted to be sensitive to PARP inhibitor (Supplementary Table S9), was sensitive to talazoparib (Fig. 5a), but resistant to olaparib (Fig. 5c). In conclusion, our model predicted the responses to PARP inhibitor of these PDTCs with 93.8% accuracy (15 out of 16) in the ex vivo validation (Fig. 5a and Supplementary Table S9), and with 100% accuracy in three independent in vivo validations (Fig. 5b–d and Supplementary Table S9).

We obtained another panel of PDTCs of ovarian cancer, where the in vivo responses to niraparib of five PDTCs had been verified in PDX mouse models.^42^ Our gene panel predicted the in vivo responses to niraparib of these models with 100% accuracy, including 2 HR-proficient models sensitive to niraparib (PH039 and PH087) (Fig. 5e and Supplementary Table S10). Curiously, the two resistant models PH095 and PH080 reportedly contain a BRCA2 mutation and a CDK12 mutation, respectively,^42^ suggesting that HR gene examination is not always a reliable readout of HR status. Indeed, recent studies indicate that mutagenic single-strand DNA gap repair by REV1-Polz-dependent translesion synthesis might be an important factor conferring resistance of BRCA1/2 mutant cells to PARP inhibitor and cisplatin.^43,44^ Despite this, all the resistant models express low level of factors in the gene panel (Fig. 5e and Supplementary Table S10), suggesting the gene panel as a reliable readout of drug response to niraparib.

Since prior studies focused on searching for biomarkers of BRCAness as an indicator for PARP inhibitor response, only mutation information was collected but not transcriptional information. Therefore, we collected a cohort of 58 ovarian cancer patients with platinum-sensitive relapsed disease who achieved objective response (partial or complete response [PR or CR]) following the last platinum-based therapy prior to Olaparib Maintenance monotherapy from multiple hospitals in China mainland, which to our knowledge might be the only clinical cohort of PARP inhibitors with both RNA-seq and whole genome sequencing data. Five patients were excluded for further analysis since they had stopped taking a PARP inhibitor before disease relapse or death. Another three patients were also excluded because of loss of contact. The average follow-up time of the final remaining 50 patients was 422.86 days, and there were in total 10 PFS events. All PARP inhibitor-treated patients are selected from platinum-sensitive patients, explaining why most of them were found to have high shallowHRD scores (Fig. 5f and Supplementary Table S11). According to multiple clinical studies on PARP inhibitor maintenance treatment for platinum-sensitive recurrent ovarian cancer patients, the median duration of progression-free survival of patients either with or without homologous recombination deficiency in PARP inhibitor group ranged from 7.4 to 13.6 months. The median duration of patients with BRCA1/2 mutations in some of these studies were beyond this range^12–15^; however, this is expected since patients with BRCA1/2 mutations are the most sensitive subpopulation to PARP inhibitor treatment. Therefore, we defined resistant patients as those who relapsed or died in less than 10 months (300 days). We performed multivariate Cox regression analysis using our gene panel and HR status. The results verified that the gene panel has statistical significance in predicting sensitivity of patients to PARP inhibitor (*P* = 0.044, *B* = −7.423, HR = 0.001), which is more significant than that of HR status (*P* = 0.089, *B* = −5.144, HR = 0.006). We further analyzed the clinical data in detail, and found that all sensitive patients have either HR deficiency or relatively high expression of genes in the panel (Fig. 5f and Supplementary Table S11). Although the shallowHRD score was unavailable for two patients, including sensitive patient P36 with low expression of genes in the panel, a BRCA2 nonsense variant report was found in her clinical record (Supplementary Table S11). Consistent with our hypothesis, the other patient without a shallowHRD score was resistant with low expression of genes in the panel. For all the patients with either normal or borderline HR function, high expression of genes in the panel could predict sensitivity to PARP inhibitor (Fig. 5f and Supplementary Table S11). Curiously, most of the PARP inhibitor-resistant patients had been profiled as HR-deficient according to their corresponding shallowHRD scores (Supplementary Table S11), suggesting that they might have acquired resistance due to prior platinum treatment. Despite this, all the resistant patients express relatively low level of genes in the panel (Fig. 5f and Supplementary Table S11). Overall, our gene panel predicts the response to PARP inhibitor of patients without apparent HR defects (borderline or HR proficiency) with 100% accuracy (Fig. 5f and Supplementary Table S11). It is worth mentioning that some previous work argued that the upregulated rRNA transcription rate caused by c-MYC overexpression/amplification might enhance PARP sensitivity.^45,46^ However, we found no correlation between the transcriptional expression of c-MYC and the PCA score of the gene panel in these PARP inhibitor-treated patients (*r* = 0.12; *P* = 0.4) (Supplementary Fig. S19h), and consequently, the expression of c-MYC cannot be used as a predictor of PARP inhibitor response (Supplementary Table S11). This suggests that the vulnerability of cellular ribosomal biogenesis to the PARP inhibitor was conferred by the upregulation of genes in the panel, but not by the c-MYC-enhanced rRNA transcription rate. These results again indicate that the gene panel might be a reliable readout of drug response to PARP inhibitor even in the real world in the clinic.

Furthermore, we treated the ovarian cancer organoids library mentioned above with olaparib. The results confirmed that high PCA score of the gene panel predicts sensitivity to PARP inhibitor, and that all the resistant samples have relatively low PCA score of the gene panel (Fig. 5g and Supplementary Table S7). Like the results for cisplatin treatment, gene mutations involved in HR repair are found in all four sensitive samples with low PCA scores of the gene panel (Fig. 5g and Supplementary Tables S7 and 8), including the mutation of *SMAD3*. SMAD3 was reported to induce resistance to PARP inhibitors via promoting DNA double-strand break repair.^40^ Interestingly, O7 is sensitive to cisplatin while resistant to olaparib (Supplementary Table S7), suggesting that there might be additional factors in O7 that differentially affect PARP inhibitor and cisplatin response. These results indicate that the combined examination of HR status and gene panel effectively screens out patients who are responders to PARP inhibitor/cisplatin.

### Listed drugs upregulating genes in the panel sensitized cells to PARP inhibitor/cisplatin without causing HR deficiency

**Fig. 6:**
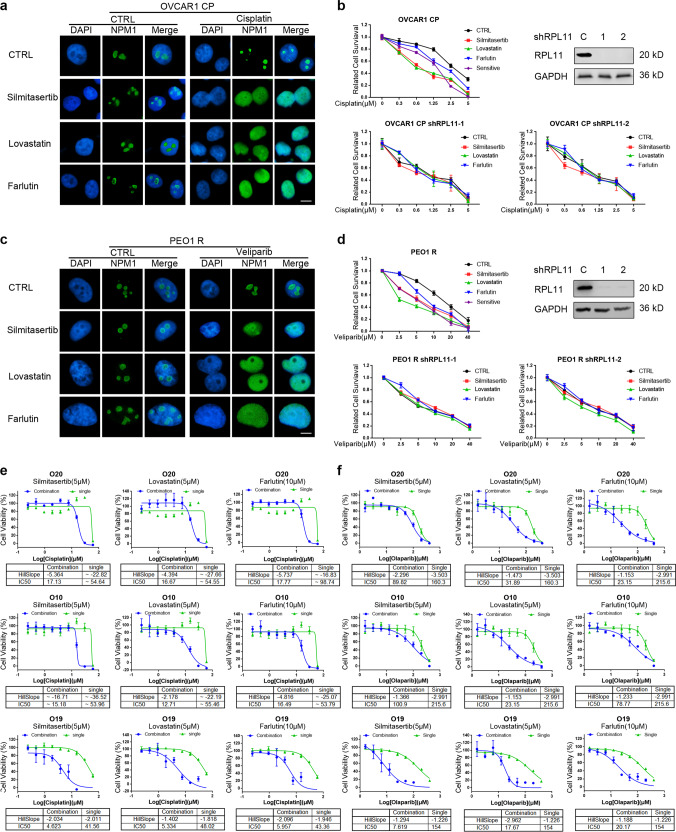
Three marketed drugs induced a novel type of synthetic lethality in samples resistant to PARP inhibitor/cisplatin. **a**, **c** Cisplatin-resistant OVCAR-1 CP (**a**) and veliparib-resistant PEO1 R (**c**) were pretreated with three indicated drugs for 24 h before treatment with veliparib or cisplatin for another 6 h (Scale bar: 5 μm). The localization of NPM1 was detected by immunofluorescence to indicate the ribosomal stress. **b**, **d** The expression of RPL11 was suppressed by lentivirus-mediated shRNA to block the lethal effects of ribosomal stress in OVCAR-1 CP (**b**) and PEO1 R (**d**). The efficient knockdown of RPL11 expression was confirmed by western blots analysis. The cells were pretreated with the three indicated drugs for 24 h. The parental sensitive OVCAR-1 or PEO1 were used as positive controls. Colony-formation assay was used to determine the drug sensitivity to cisplatin (**b**) or veliparib (**d**). **e**, **f** Cell viability assay was performed for cisplatin (**e**) or Olaparib (**f**) treated ovarian cancer organoids with/without pretreatment with the listed drugs

We previously found several listed drugs synergize with PARP inhibitor/cisplatin without causing HR efficiency, Farlutin (Progestin), Lavastatin (HMG-CoA-reductase inhibitor) and Silmitacertib (CK2 inhibitor); however, the underlying mechanism was unclear. Surprisingly, we found that all of them induced remarkable upregulation of the factors in the gene panel in cell lines with original low-expressed genes of the gene panel (Supplementary Fig. S20a). We then adopted these three listed drugs to pre-treat two cell lines (PEO-1R and OVCAR-1 CP) with acquired resistance to PARP inhibitor and cisplatin, respectively. All of them significantly upregulated the expressions of the factors in the gene panel in these two resistant cell lines (Supplementary Fig. S20b, c) without causing significant alterations in both HR efficiency (Supplementary Fig. S21a, b) and cell cycle, except Lavastatin that caused mild G1 arrest in both cell lines (Supplementary Fig. S21c, d). Interestingly, PARP inhibitor and cisplatin-induced ribosomal stress in these pretreated resistant cell lines by these three drugs, but not in those without pretreatment of these drugs (Fig. 6a, c). Most importantly, these original resistant cell lines became sensitive to PARP inhibitor/cisplatin after being pretreated by these drugs, and were eliminated via ribosomal stress, since RPL11 knockdown effectively blocked the sensitization effects of these three list drugs (Fig. 6b, d). Especially, Silmitacertib and Lavastatin induced equivalent or even stronger responses of these resistant cell lines to PARP inhibitor or cisplatin compared with their corresponding original sensitive cell lines (PEO1 and OVCAR-1), respectively (Fig. 6b, d). We further adopted these three chemicals on three intrinsically resistant models to both olaparib and cisplatin from our ovarian cancer organoids library. The pretreatment of these chemicals also significantly sensitized these original resistant organoid models to cisplatin and PARP inhibitor, respectively (Fig. 6e, f and Supplementary Fig. S22).

In conclusion, these three repositioning drugs hold the potential to further extend the clinical application of PARP inhibitor/cisplatin in treatment-resistant patients. In addition, these results also imply that modulating the gene panel expression might be a parallel and alternative therapy strategy to HR deficiency in patients resistant to PARP inhibitor/cisplatin, although it still needs further verification and more in-depth study to search for potential additional requirements for this therapy strategy besides upregulating the gene panel.

## Discussion

Most prior studies have focused on searching for biomarkers that indicate BRCAness for PARP inhibitor/cisplatin response. Our study moves in a different direction by proposing a new signature for PARP inhibitor/cisplatin response for HR-proficient patients and implies a potential strategy to induce synthetic lethality with these drugs. Our study for the first time constructs a gene panel as the predictive biomarker of PARP inhibitor/cisplatin for HR-proficient patients. We postulated that the gene panel can complement HR status, and propose to combine the examination of these two indicators. Specifically, patients with high expression of genes in this gene panel can be screened out for PARP inhibitor/cisplatin treatment; whereas, other patients need to be examined for their HR status (Supplementary Fig. S18). As gene panel testing is much cheaper than HR status testing, checking the gene panel first would be faster and more cost-effective, and it can exempt many patients from expensive HR status detection. This novel combined examination was verified to be highly reliable by multiple published clinical datasets, as well as an ovarian cancer organoids library we established. More importantly, the combined test demonstrated superior predictive value in the cohort of 50 PARP inhibitor-treated ovarian cancer patients that we collected. Although PARP inhibitors and cisplatin are classified together in the current study, the frequently observed differences in patient sensitivities to these drugs, suggest that there are different thresholds for the gene panel and the HR status in cancer patients (if quantified by i.e., COSMIC signature 3, ShallowHRD or HRD score, etc.). Therefore, we propose a new paradigm that extends the use of PARP inhibitors/cisplatin in the clinic, which will allow more patients without HR defects to be treated with these drugs (Supplementary Fig. S18). However, due to the heterogeneity of ovarian cancer and the inherent bias of the retrospective design, further validation in a more rigorous prospective study will be required before applying this new paradigm in cancer precision diagnosis and therapy.

Interestingly, our results suggest that the gene panel is a more reliable readout than HR status. In fact, across five independent clinical trials, many BRCA-mutant patients still fail to respond to PARP inhibitor treatment, with an average objective response rate of 47%,^47^ The non-responding BRCA-mutant patients may be explained by phenomena including concurrent mutations such as loss of either TP53BP1^48^ or PTEN^49^ that can lead to restoration of HR-mediated DNA repair. However, in our patient cohort, all of the patients with high expression of genes in the gene panel responded well to PARP inhibitor, and other validation data sets reached similar conclusions. Therefore, this gene panel holds deep potential to become a reliable biomarker and guide precision cancer therapy in the future. The small size of the gene panel and its near perfect compatibility with current clinic HR status test favor its broad-scale clinical application.

Furthermore, we also reveal the underlying mechanism of the new synthetic lethality related to the gene panel. It is generally accepted that cell death during PARP inhibitor/cisplatin treatment is mainly caused by failure of DNA replication/repair. We think this is still the case in HR-deficient cells with low expression of genes in the panel. Unexpectedly, PARP inhibitors and cisplatin also share a common central mediator of cytotoxicity in HR-proficient cells, the DNA damage signaling-induced IRBC. This represents a new synthetic lethal target of these drugs in HR-proficient tumors. This finding connects two previous important studies,^4,50^ the prior one argued that cisplatin kills cells by DNA damage but without inducing ribosomal stress,^4^ while the other one inferred that PARP inhibitors might directly deter ribosomal function.^50^ We cannot rule out the possibility that PARP inhibitor and cisplatin might stress other cellular activities or functions, which still needs further study.

Based on our results, we put forward a new model that determines cell fate during PARP inhibitor/cisplatin treatment (Fig. 3h), in which ATM signaling simultaneously controls pro-apoptotic ribosomal stress and pro-survival HR repair to balance cell fate. HR status is an indicator of the cellular capability to repair specific lethal DNA damage, and the gene panel is the indicator of IRBC-dependent apoptosis caused by overall high level of DNA damage. Thus, we speculate that this model might constitute an evolutionary strategy to prevent mutagenesis: some cells repair specific lethal DNA damage for survival, while some cells are sacrificed to prevent mutagenesis when the accumulated DNA damage exceeds the cellular repair capability. The double-edged sword effect of the ATM signaling also provides a possible explanation for the conflicting results in multiple clinical studies regarding the synthetic lethality of ATM mutation/inhibition with PARP inhibitor/cisplatin.^32–37^

Lastly, our study proposes three marketed drugs that upregulate the expression of genes in the panel without causing HR deficiency; all of which synergize with PARP inhibitor/cisplatin via inducing ribosomal stress. These repurposing drugs have revealed potential to further extend the clinical application to PARP inhibitor/cisplatin treatment-resistant patients. However, their potential side effects upon the combination with PARP inhibitor/cisplatin need to be taken into consideration in the future. This finding supports the potential of this gene panel for precise prediction of tumor response to PARP inhibitor/cisplatin from another angle. More importantly, it implies that modulating the expression of genes in the panel might represent a parallel and alternative therapeutic strategy for patients resistant to PARP inhibitor/cisplatin. There have been intense efforts to discover more genes involved in HR repair as targets to help develop new drugs synergizing with PARP inhibitors/cisplatin. However, to the best of our knowledge, no marketed drugs have been found by this traditional strategy. Therefore, our results could lead to a new direction, searching for marketed drugs that upregulate the expression of genes in the gene panel in the future. This new mechanism will need further verification and more in-depth studies to understand additional requirements for this novel therapy strategy besides upregulating the expression of genes in the panel.

In summary, our study discovers a new biomarker and mechanism of action of PARP inhibitor/cisplatin, and proposes an extended clinical use of PARP inhibitors and cisplatin.

## Materials and methods

### Data preprocessing for human cancer cell line analysis

Drug sensitivity data for human cancer cell lines were obtained from the Genomics of Drug Sensitivity in Cancer (GDSC) data,^17^ and the read count of RNA-seq profile of cell lines was available from the Cancer Cell Line Encyclopedia (CCLE).^16^ All cell lines with both RNA-seq profile in CCLE and drug sensitivity data in GDSC were used to perform the following pan-cancer analysis. Only coding genes were selected from the RNA-seq data. Unrecognized genes as well as genes whose expression for all samples was zero were excluded. For genes with multiple records, we calculated the sum of the expression levels of their multiple records as their expression level for the subsequent weighted correlation network analysis (WGCNA) and Pearson correlation analysis. The remaining RNA-seq data was subjected to the variance-stabilizing transformation (VST) function from DESeq2 prior to downstream analysis. Both the drug sensitivities data and VST-transformed gene expression were regressed on annotations describing tissue of origin and cancer subtype (“ccle_primary_site”, “ccle_primary_hist”, “ccle_hist_subtype_1”) using linear regression. Then the drug sensitivities data were Z-scaled.

### WGCNA analysis for cell lines data

We constructed the signed co-expression network via WGCNA R package^51^ using coding gene-expression levels after preprocessing. We calculated the Pearson correlation coefficients between the eigengene of each gene module and the drug sensitivity (ln(IC_50_)) after preprocessing. The co-expressed gene module that most negatively correlated with IC_50_ constituted drug “signatures”. The genes in the signature module of these drugs derived by WGCNA were used to perform GO enrichment analysis via Gene Ontology enRIchment anaLysis and visuaLizAtion tool (Gorilla).^52^

### Pearson’s correlation analysis for cell lines data

Pearson correlation coefficients between drug sensitivity (ln(IC_50_)) and gene-expression levels after data preprocessing were calculated and then Z-scaled. The false discovery rate (FDR) estimation was performed from the calculated p values by qvalue R package.^53^ Gene set enrichment analysis (GSEA) was performed on the drug sensitivity–gene-expression correlations by WebGestalt using pathway annotations from the Kyoto Encyclopedia of Genes and genomes (KEGG).^54^

### COSMIC mutational signature analysis

The mutect-generated mutation data were obtained from TCGA database (http://tcga-data.nci.nih.gov/tcga/). The mutational signature analysis was performed using latest R packages “MutationalPatterns”,^55^ which deciphered signatures of mutational processes from mutational catalogs of cancer genomes.^56^ The COSMIC mutation signature 3, whose proposed etiology was failure of DNA double-strand break repair by homologous recombination, was used as HR Defect signature (https://cancer.sanger.ac.uk/cosmic/signatures_v2).

### Prediction of cellular drug response

Drug sensitivity data for cancer cell lines were downloaded from GDSC database,^17^ and the expression levels of all the biomarkers in our gene panel were available from CCLE database.^16^ The PCA score^6^ was calculated based on the expressions of the genes in the gene panel to predict the sensitivity of these cell lines (“high” versus “low” based upon a median cutoff value). The HR status of cell lines was determined by their levels of COSMIC mutation signature 3 published in ref. ^57^ Breast cell lines with HR defect signature levels below the first quartile of all the breast cancer cell lines with quantification of HR signature (95.46113286) were defined as normal HR function group. Ovarian cell lines with HR defect signature levels below the lower tertile of all the ovarian cancer cell lines with quantification of HR signature (115.8824107) were defined as normal HR function group. For combined analysis of cell lines from all tumor types, the tissue-specific differences for both gene-expression and drug sensitivity data were removed via a linear regression model as described above. *P* values were calculated using one-sided *t* tests.

### Cell lines and cell culture conditions

HEK293T, OV90, HCT116, HT-29, ZR75-30, HCC1954 and BT549 were purchased from American Type Culture Collection (ATCC). OVKATE was purchased from JCRB Cell Bank. OV56, A2780 and IGROV1 were purchased from Sigma. COLO678 was purchased from Leibniz Institute DSMZ. Human ovarian PARP inhibitor and cisplatin-resistant cancer cell lines, PEO1/ABT-888 and OVCAR-1/Cisplatin and their parental cell lines, were gifts from Prof. Scott Kaufmann (Department of Oncology, Mayo Clinic). Cells were maintained under recommended culture conditions. PEO1/ABT-888 and OVCAR-1/Cisplatin were cultured in the media supplemented with 10 μM ABT-888 and 0.5 μg cisplatin, respectively. Cells were regularly tested for mycoplasma contamination.

### Cell cycle analysis

Cells were harvested and fixed in a single-cell suspension in 70% cold ethanol. Cells were then incubated at −20 °C overnight and stained the next day with propidium iodide (PI) containing RNase for 4 h at 4 °C. Cell cycle was then analyzed by flow cytometry and ModFit LT software.

### Immunofluorescence microscopy

Indicated cells were cultured on coverslips and treated with 50 µM cisplatin (Sigma, P4394), 100 µM olaparib (LC Laboratories, O-9201), 10 µM DNA-PK inhibitor AZD7648 (ChemScene, CS-0091859), 10 µM ATM inhibitor KU55933 (Abcam, ab120637) or 10 µM ATR inhibitor VX970 (Selleckchem, S7102) for 6 h. After washing with PBS, cells were fixed in 3% paraformaldehyde for 15 min and permeabilized in 0.5% triton X-100 solution for 5 min at room temperature. Cells were then blocked with 5% goat serum and incubated with indicated primary NPM1 (Invitrogen, 32-5200) or γH2AX (CST, 9718 S) antibody overnight in 4 °C. Subsequently, samples were washed and incubated with Alexa Flour labeled secondary antibody for 60 min. DAPI staining was performed to visualize nuclear DNA. The coverslips were mounted onto glass slides with anti-fade solution and visualized using a Nikon ECLIPSE E800 fluorescence microscope. For OVCAR-1 CP cells and PEO-1R cells, they were pretreated with lovastain (5 μM), farlutin (5 µM) or slinitasertib (5 μM) for 36 h, followed by 50 µM cisplatin (Sigma, P4394) and 100 µM olaparib (LC Laboratories, O-9201) for 6 h, respectively. Then they were analyzed by immunofluorescence staining and confocal imaging as mentioned above.

### Ribosome fractionation

BT549 cells were plated into 15 cm dishes at a density of 90%. The cells were treated with 50 µM cisplatin, 100 µM olaparib or 10 nM ATM inhibitor KU55933 for 6 h, washed three times with ice cold PBS, and scraped gently into 1.5 mL Buffer A (250 mM sucrose, 250 mM KCl, 5 mM MgCl_2_, 50 mM Tris-HCl, pH 7.5 and supplemented with 1× protease inhibitors, PMSF, NaF, β-glycerophosphate and Aprotinin. IGEPAL-30 was added to a final concentration of 0.7% (v/v) and incubated on ice for 20 min with frequent mixing. Five percent of the lysate was separately stored and used for input of whole cell extract. The remaining lysates were centrifuged at 12,500 RCF for 10 min. The protein concentration of the lysates was equilibrated using Buffer A and the KCl levels were adjusted to 500 mM with 3 M KCl. The lysates were loaded onto 2.5 mL of sucrose cushion (1 M sucrose, 0.5 M KCl, 5 mM MgCl_2_ and 50 mM Tris-HCl pH 7.5) in polypropylene tubes (Beckman Coulter, 328874). The tubes were centrifuged for 4 h at 45,000 rpm in a Beckman coulter ultracentrifuge (Optima L-80 XP) using a SW60Ti rotor. After the spin, the ribosomal pellets were re-suspended in 1× loading Buffer. The samples were used for western blot to detect the expression of RPL10a (Abcam, ab174318) and RPL26 (Sigma-Aldrich, HPA030449). GAPDH (CST, 2118 S) was used for internal reference.

### Proliferation assays and colony-formation assays

For MTS, assays were performed using the CellTiter 96 Aqueous One Solution Cell Proliferation Assay kit (Promega, Madison, WI, USA). Cells (2 × 10^3^/100 µl per well) were plated on 96-well culture plate and treated with indicated Drugs for 3 days for each experimental condition. Each well was incubated with 20 µl of MTS reagent for 1–4 h at 37 °C in a humidified, 5% CO_2_ atmosphere. The absorbance was corrected relative to blank wells containing MTS reagent and media only. The MTS readings at specified time points were normalized to vehicle controls readings. For colony-formation assay, indicated cells were seeded in triplicate in 6 wells culture plate (600 cells per well). After adherence, cells were treated with PARP inhibitor or cisplatin in indicated concentration. After 10–14 days, media was removed, and plates were rinsed carefully with PBS three times. Macroscopic colonies were stained with Coomassie stain (210 mL H_2_O, 210 mL MeOH, 85 mL acetic acid, 0.5 g Coomassie Blue) 30 min. Quantification was done manually. Data was normalized to vehicle control. For experiments in resistant cells, OVCAR-1 CP cells were pretreated with lovastatin (selleckchem, S2061) (5 μM), farlutin (TargetMol, T1261) (10 μM) or slinitasertib (selleckchem, S2248) (2 μM) for 36 h and PEO-1R cells received lovastatin (1 μM), farlutin (10 μM) or slinitasertib (5 μM) treatment for 36 h. Cells were then treated with ABT-888 or cisplatin in indicated concentration, respectively. After 12–14 days, colonies were fixed and quantified.

### Real-time PCR and lentiviral infection

Total RNA was extracted with TRIzol (Invitrogen, 15596026). The PrimeScript RT Reagent Kit (TaKaRa, RR037A) was used for cDNA synthesis. Real-time PCR was carried out using the ABI Prism 7300 Sequence Detection System (Applied Biosystems, Foster City, CA). Data normalization was accomplished using the endogenous control GAPDH, and the normalized values were subjected to a 2 − ΔΔCt formula Statistical analyses. All the shRNA plasmids were obtained from Sigma. The shRNA lentiviral particles were packaged and transduced into the indicated cells according to the manufacturer’s guidelines. The RPL11 knockdown efficiency was verified by immunoblot with RPL11 antibody (Proteintech, 16277-1-AP). The RT-PCR primer and the shRNA guide sequences are provided in Supplementary Table S12.

### DNA repair assay

Integrated DNA repair reporter systems were used to determine the HR efficiency. Briefly, indicated cells co-transfected with reporter DR-GFP and I-SceI expression vector (pCBA-I-SceI). Cells were harvested two days after I-SceI transfection and subjected to flow cytometric analysis to examine the percentage of GFP-positive cells. Results were normalized to wild-type cells.

### Survival analysis for clinical patients treated with cisplatin

The clinical data of ovarian cancer (OV) were obtained from TCGA database. The information of patients treated with cisplatin is available from “nationwidechildrens.org_clinical_drug_ov.txt”. The survival information of these ovarian cancer patients, including vital status, tumor status, etc., is available from “nationwidechildrens.org_clinical_follow_up_v1.0_ov.txt” (*n* = 113). The new event information is available from “nationwidechildrens.org_clinical_follow_up_v1.0_nte_ov.txt”. We performed overall survival, progressive-free interval, and disease-free interval analyses for these patients. Patients with levels of COSMIC mutation signature 3 above the first quartile were defined as HR defect group and the other patients were defined as normal HR function group. The RPKM counts of RNA-Seq data for all cancer types were obtained from TCGA database (http://tcga-data.nci.nih.gov/tcga/). The PCA score^5,6^ was calculated based on the expressions of the genes in the gene panel to quantify the overall rank of the gene panel (“high” versus “low” based upon a median cutoff value). In total, 84 cisplatin-treated patients with both whole-exome sequencing and RNA-seq data were used to perform these analyses. Permutation tests (1000 times) were carried out to compare the performance (hazard ratio) of randomly selected lists of 8 genes with our gene panel and derive an empirical *P* value determined by one-tailed test. The microarray data and survival information of patients from the Hennessy cohort treated by cisplatin were obtained from the work of McGrail and colleagues.^39^ There are in total 87 platinum-treated patients in this cohort, in which 21 patients are treated with cisplatin. The PCA score^6^ was calculated based on the expression of genes in the gene panel to predict the sensitivity of these PDTCs (“high” versus “low” based upon a median cutoff value). Both overall survival and progression-free survival analysis were performed based on these patients.

### Patient-derived tumor xenografts (PDTXs)

The sensitivity to talazoparib/olapatrib of a panel of PDTX-derived tumor cells (PDTCs) of breast cancer was acquired from the work of Bruna and colleagues.^41^ The relevant gene-expression and mutation data were downloaded from BCaPE (Breast Cancer PDTX Encyclopaedia) https://caldaslab.cruk.cam.ac.uk/bcape/. The PCA score was calculated based on the expression of genes in the gene panel to quantify the overall rank of the gene panel (“high” versus “low” based upon a median cutoff value).^6^ *P* values were calculated using one- tailed *t* tests.

### Establishment of ovarian cancer organoid cultures

OV organoids were derived from surgery samples or ascites of ovarian cancer patients at Yixing People’s Hospital, Yixing, China. The study received approval from the Ethical Committee of Yixing People’s Hospital (Trial No. 060, 2020). Briefly, tumor tissue was minced with scissors and dissociated with 5 mL of 5 mg/mL collagenase type II (Sigma-Aldrich) to generate single-cell suspension. The cell pellet was collected after centrifuging at 400×*g* for 3 min and suspended in 50% (v/v) cold growth factor reduced Matrigel (BD Biosciences). Drops of 200 µl of Matrigel cell suspension were allowed to solidify on pre-warmed 24-well ultra-low binding culture plate (Corning 3471) at 37 °C for 30 min. Upon Matrigel stabilization, 500 μl of modified OV organoid medium (K2O-OKL-OA) was added, and plates were transferred to humidified 37 °C, 5% CO_2_ incubators. Medium was changed every 3–4 days and organoids were passaged every 1–2 weeks.

### Transcriptomic profiling and DNA sequencing of OV organoids

RNA was isolated from organoids with TRIzol (Invitrogen). RNA libraries were generated with the NEBNext® Ultra™ RNA Library Prep Kit for Illumina. Libraries were multiplexed and paired-end sequenced (2 × 75 bp) on Illumina NextSeq. RNA-seq data were processed with Novagene RNA analysis pipeline (assigned accession number: HRA001499). Gene features without corresponding gene symbols and with duplicate mappings were removed. For DNA sequencing, organoids were pelleted and DNA was isolated using a DNeasy blood and tissue kit (QIAGEN). Sequencing was performed using an Illumina HiSeq system for 673 tumor-related genes in WuXi NextCODE at Shanghai, China.

### Cell viability assay of ovarian cancer organoids

For cell viability assessment, organoids were dissociated into single cells, counted, and plated in Matrigel-coated 96-well plates (8000 cells per well) in triplicate for 48 h prior to drug treatment. All compounds were purchased from MCE Shanghai and dissolved in DMSO, except cisplatin dissolved in pure water. Organoids were treated with serial diluted concentrations of Olaparib or Cisplatin (1.56–400 μM and 0.23–60 μM, respectively) for 120 h before conducting to CellTiter Glo 3D viability assay. Drug-response curves were graphed and IC_50_ values were calculated using Graphpad Prism 6.0. Representative brightfield photo were taken after drug treatment. Dural drug-sensitize assays were carried out with cell viability assay as described above. Briefly, organoids were incubated with serial diluted Olaparib or Cisplatin with or without the existence of Silmitasertib (5 μM), Lovastatin (5 μM), or Farlutin (10 μM), respectively. After 120 h of treatment, cell viability of ovarian cancer organoids was evaluated and Drug-response curves were graphed and IC_50_ values were calculated with Graphpad Prism 6.0.

### Progression-free survival analysis for ovarian cancer patients treated with PARP inhibitor

The observational study was approved by the Ethical Review Committee of respective hospitals (ethical batch number: 2021S031; 20180186). A retrospective review of 58 platinum-sensitive relapsed ovarian cancer patients who received PARP inhibitor therapy from August 2018 to July 2021 in hospitals of mainland China was conducted. Progression-free survival analysis was performed. All the cases meeting the inclusion criteria were collected to address potential sources of bias. Diagnostic criteria were determined by the clinicians. Inclusion criteria were as follows: (1) patients, 18 years of age or older, had relapsed high-grade serous or high-grade endometrioid epithelial OC, including primary peritoneal and/or fallopian tube cancer; (2) patients had received at least two previous lines of platinum-based chemotherapy and achieved objective response (partial or complete response [PR or CR], according to modified Response Evaluation Criteria in Solid Tumors [RECIST] version 1.1); (3) patients maintained with PARP inhibitor monotherapy; (4) samples with complete information and are traceable to the original records; (5) patients with only one primary cancer; (6) patients retaining surgical specimens from which RNA data can be extracted. Exclusion criteria were as follows: (1) patients undergoing other adjuvant therapies such as radiotherapy and chemotherapy during PARP inhibitor maintenance treatment; (2) patients had stopped taking PARP inhibitor before disease relapse or death; (3) patients losing contact. Based on the above criteria, five patients were excluded for further analysis since they stopped taking PARP inhibitor before disease relapse or death. Another three patients were also excluded because of losing contact. Finally, a total of 50 samples were sent for sequencing, including both RNA-seq and whole Genome sequencing. The raw sequencing data was deposited in the Genome Sequence Archive in BIG Data Center, Beijing Institute of Genomics, Chinese Academy of Sciences (https://bigd.big.ac.cn/gsa-human/browse/, assigned accession number: HRA001031 and HRA001069). Eighteen of these patients were telephone followed up until May, 2021 (HRA001069), and the remaining 32 patients were followed up to April, 2021 (HRA001031) with a medium follow-up time of 340.5 days. The progression-free survival analysis was performed for all these 50 patients. The PFS events includes disease progression or death from any cause. The evaluation indexes of recurrence included CA125 test, imaging evidence, and clinical diagnosis of recurrence. Clinicians judged whether the patient relapses according to the RECIST 1.1 (Response Evaluation Criteria in Solid Tumors RECIST Version 1.1) combined with the dynamic rise of CA125. In addition, according to multiple clinical studies on PARP inhibitor maintenance treatment for platinum-sensitive recurrent ovarian cancer patients, the medium duration of progression-free survival of patients either with or without homologous recombination deficiency in PARP inhibitor group ranged from 7.4 to 13.6 months. The medium duration of patients with BRCA1/2 mutations in some of these studies were beyond this range,^12–15^ which is expected since patients with BRCA1/2 mutations are the most sensitive subpopulation to PARP inhibitor treatment. Therefore, we defined resistant patients as those who relapsed or died in less than 10 months (300 days). Based on the transcriptional expression of genes in the gene panel from the RNA-seq data, a PCA score^6^ was calculated and the overall rank of the gene panel was then quantified (“high” versus “low” was defined upon a median cutoff value). ShallowHRD^11^ can evaluate the HR status of ovarian cancer patients according to the number of large-scale genomic alterations (LGA), using only the whole genome sequencing results of tumor tissues. ShallowHRD^11^ was subsequently performed, and samples were annotated as non-HR deficiency (LGA < 15), borderline (15 ≤ LGA ≤ 19), or ‘HR deficiency’ (LGA > 19).^11^ There are two patients failed to get a ShallowHRD score due to unknown errors, and the HR status of one of them (P36) is determined by the BRCA mutation test.

### Statistical information

Survival curves and Foci statistics are presented as the mean ± SD. *P* values were calculated by one-tailed *t* tests. * and ^#^*P* < 0.05; ** and ^##^*P* < 0.01; *** and ^###^*P* < 0.001.

## Supplementary information

Supplementary materials

Table S2. The signature module for each drug derived by WGCNA analysis

Table S3. Pearson correlation analysis between basal gene expression and drug sensitivity of PARP inhibitors and cisplatin

Table S5. The prediction on response of ovarian cancer cell lines to PARP inhibitors and cisplatin

Table S6. The prediction on response of breast cancer cell lines to PARP inhibitors and cisplatin

Table S9. The prediction on response of a panel of primary breast cancer PDTX-derived tumor cells (PDTCs) to PARP inhibitor

Table S10. The prediction on response of a panel of primary ovarian cancer PDTCs to PARP inhibitor

Table S11. The prediction on response of patients with platinum-sensitive relapsed ovarian cancer treated with PARP inhibitor maintenance monotherapy

## Data Availability

The authors declare that the data supporting the findings of this study are available within the paper and its Supplementary Information.

## Code availability

The computer code is available at GitHub (https://github.com/chaohz/code-for-gene-panel-analysis.git).

## Study approval

The OV organoids were derived from surgery samples or ascites of ovarian cancer patients at Yixing People’s Hospital, Yixing, China. The study received approval from the Ethical Committee of Yixing People’s Hospital (Trial No. 060, 2020). The observational study of platinum-sensitive relapsed ovarian cancer patients who received PARP inhibitor therapy has been approved by the Ethical Review Committee of different hospitals (ethical batch number: 2021S031; 20180196).
