# Supplementary material for: A ribosomal gene panel predicting a novel synthetic lethality in non-BRCAness tumors
Source: Signal Transduct Target Ther. 2023 May 10;8:183. doi: 10.1038/s41392-023-01401-y (PMC10170152; doi:10.1038/s41392-023-01401-y)
Supplement: Supplementary file 1 — Supplementary materials [file 41392_2023_1401_MOESM1_ESM.docx]

Supplementary Materials for

**A Ribosomal Gene Panel Predicting a Novel Synthetic Lethality in non-BRCAness Tumors**

**Running title: A predictive biomarker for PARP inhibitor/cisplatin**

Chao Zhang^1,2#^, Qiang Guo^3#^, Lifeng Chen^4,5#^, Zheming Wu^2^, Xiao-Jian Yan^6^, Chengyang Zou^6^, Qiuxue Zhang^7^, Jiahong Tan^8^, Tian Fang^8^, Qunxian Rao^9^,Yang Li^10^, Shizhen Shen^11^, Min Deng^2^, Liewei Wang^12^, Huanyao Gao^12^, Jia Yu^12^, Hu Li^12^, Cheng Zhang^12^, Somaira Nowsheen^13^, Jake Kloeber^2^, Fei Zhao^2^, Ping Yin^2^, Chunbo Teng^14^, Zhongqiu Lin^9^, Kun Song^15^, Shuzhong Yao^16^, Liangqing Yao^17^, Lingying Wu^18^, Yong Zhang^19^, Xiaodong Cheng^10,20*^, Qinglei Gao^8*^, Jian Yuan^21,22*^, Zhenkun Lou^2*^, Jin-San Zhang^23,24*^

**Correspondence to:**

Xiaodong Cheng, chengxd@zju.edu.cn

Qinglei Gao, qingleigao@hotmail.com

Jian Yuan, yuanjian229@hotmail.com­­­

Zhenkun Lou, Lou.zhenkun@mayo.edu

Jin-San Zhang, Zhang_jinsan@WMU.edu.cn

**This PDF file includes:**

Figures. S1 to S22

Tables S1, S4, S7, S8 and S12

Captions for Tables S2 to S3, S5 to S6, and S9 to S11

**Other Supplementary Materials for this manuscript include the following:**

Tables S2 to S3, S5 to S6, and S9 to S11


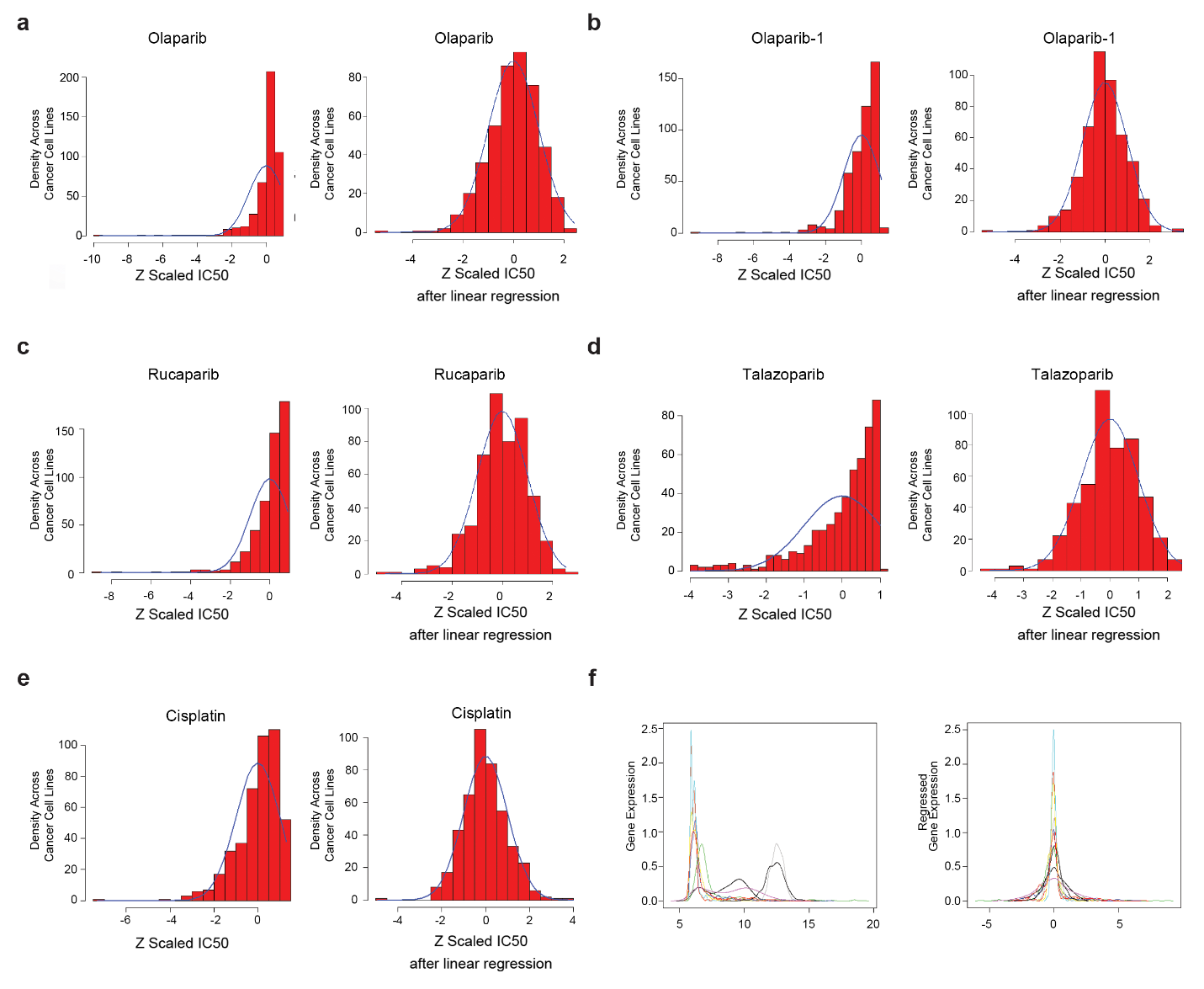


Figure S1.

Drug sensitivity data of PARP inhibitors/cisplatin from GDBC and basal gene expression of cell lines from CCLE. Distribution of (a) olaparib, (b) olaparib-1, (c) rucaparib, (d) talazoparib, and (e) cisplatin drug sensitivities across pan-cancer cell lines profiled by both CCLE and GDBC studies, quantified by their Z-scaled ln(IC_50_) before and after regressing out lineage-specific effects (*n*=444, 478, 493, 485, 444). (f) Distribution of representative VST-transformed basal gene expression across pan-cancer cell lines before and after regressing out lineage-specific effects.


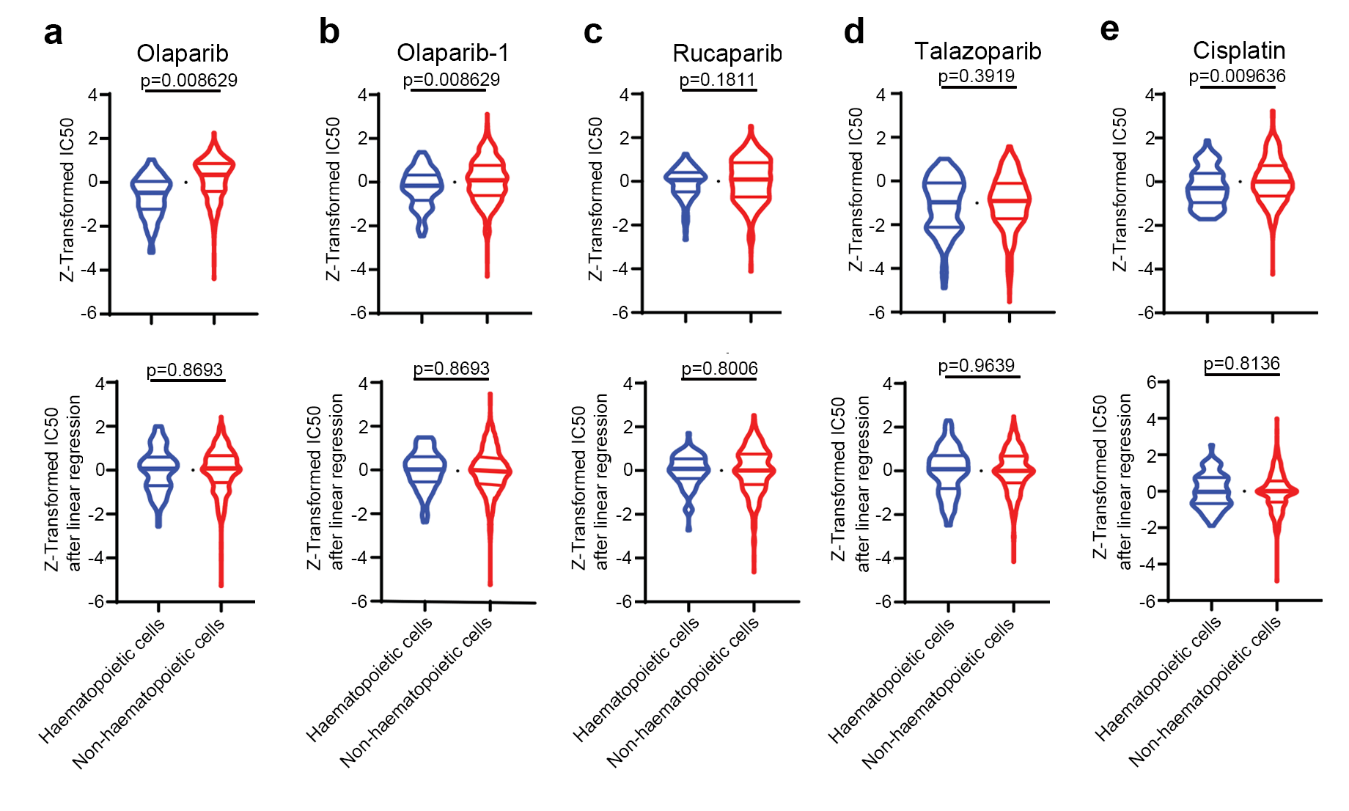


Figure S2.

The effects of regressing lineage-specific annotations on removing tissue specific of drug sensitivities of (a) olaparib, (b)olaparib-1, (c) rucaparib, (d) talazoparib, and (e) cisplatin between hematopoietic and non-hematopoietic cells (*n* = 444, 478, 493, 485, 444). Wilcoxon rank sum test.


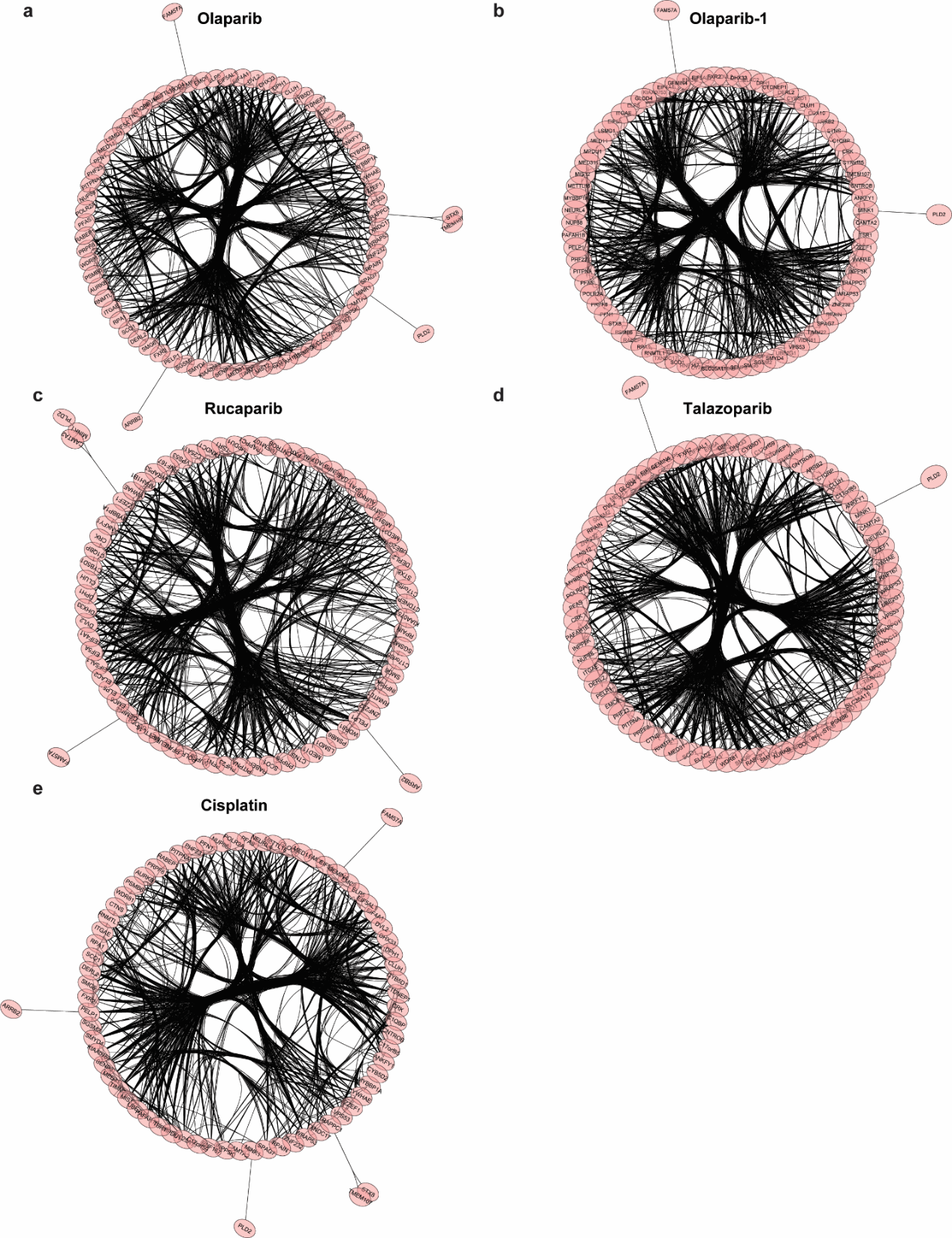


Figure S3.

The co-expressed gene modules that most negatively correlated with drug sensitivity of PARP inhibitors/cisplatin derived from WGCNA analysis, which constituted “signatures” for these drugs (a) olaparib, (b) olaparib-1, (c) rucaparib, (d) talazoparib, and (e) cisplatin. The signature modules for all the drugs in Fig. 1b are listed in Supplementary Table S2.


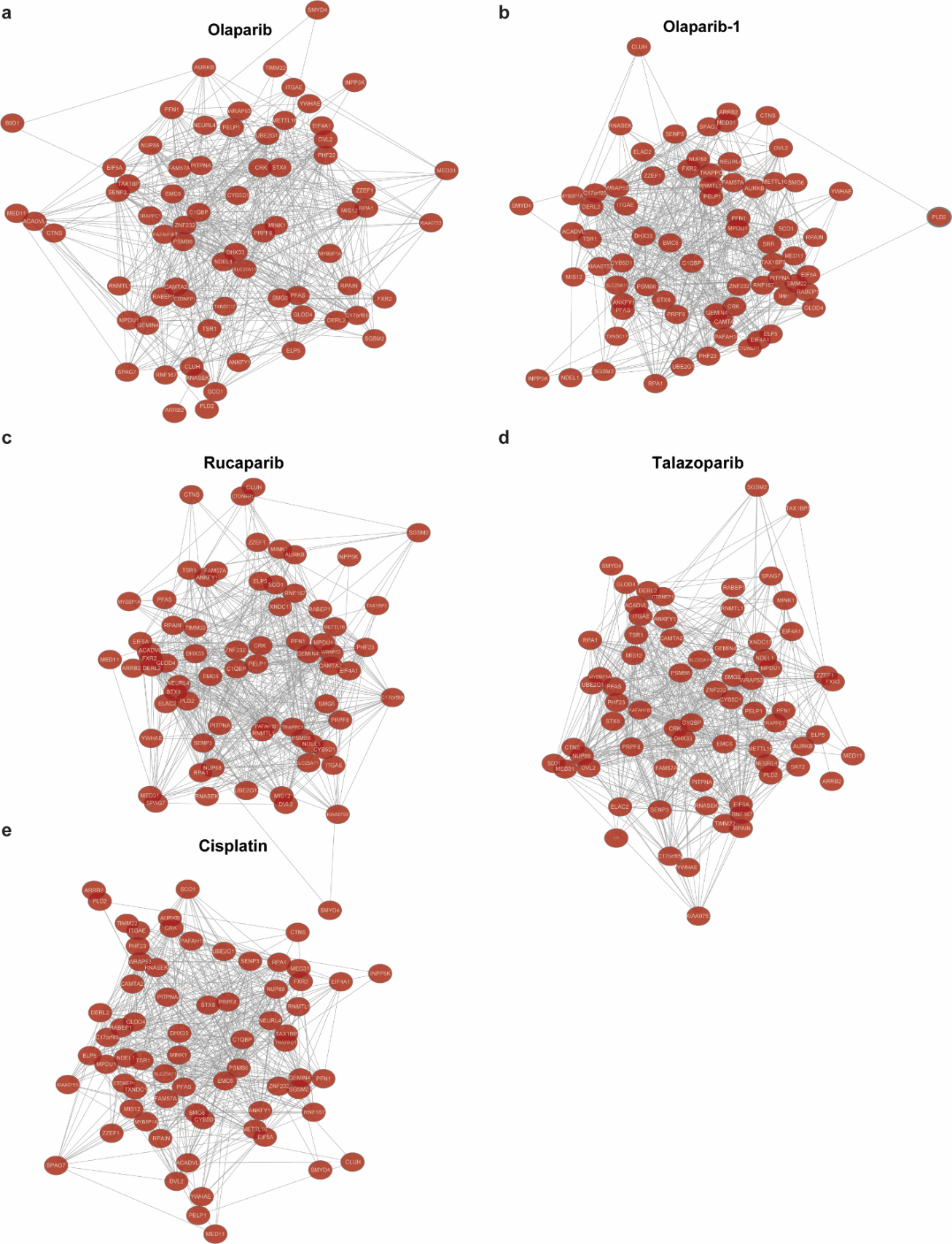


Figure S4.

The corresponding co-expressed genes of signature module for each drug involved in the human gene co-expression network established by Coexpedia. (a) olaparib, (b) olaparib-1, (c) rucaparib, (d) talazoparib, and (e) cisplatin.


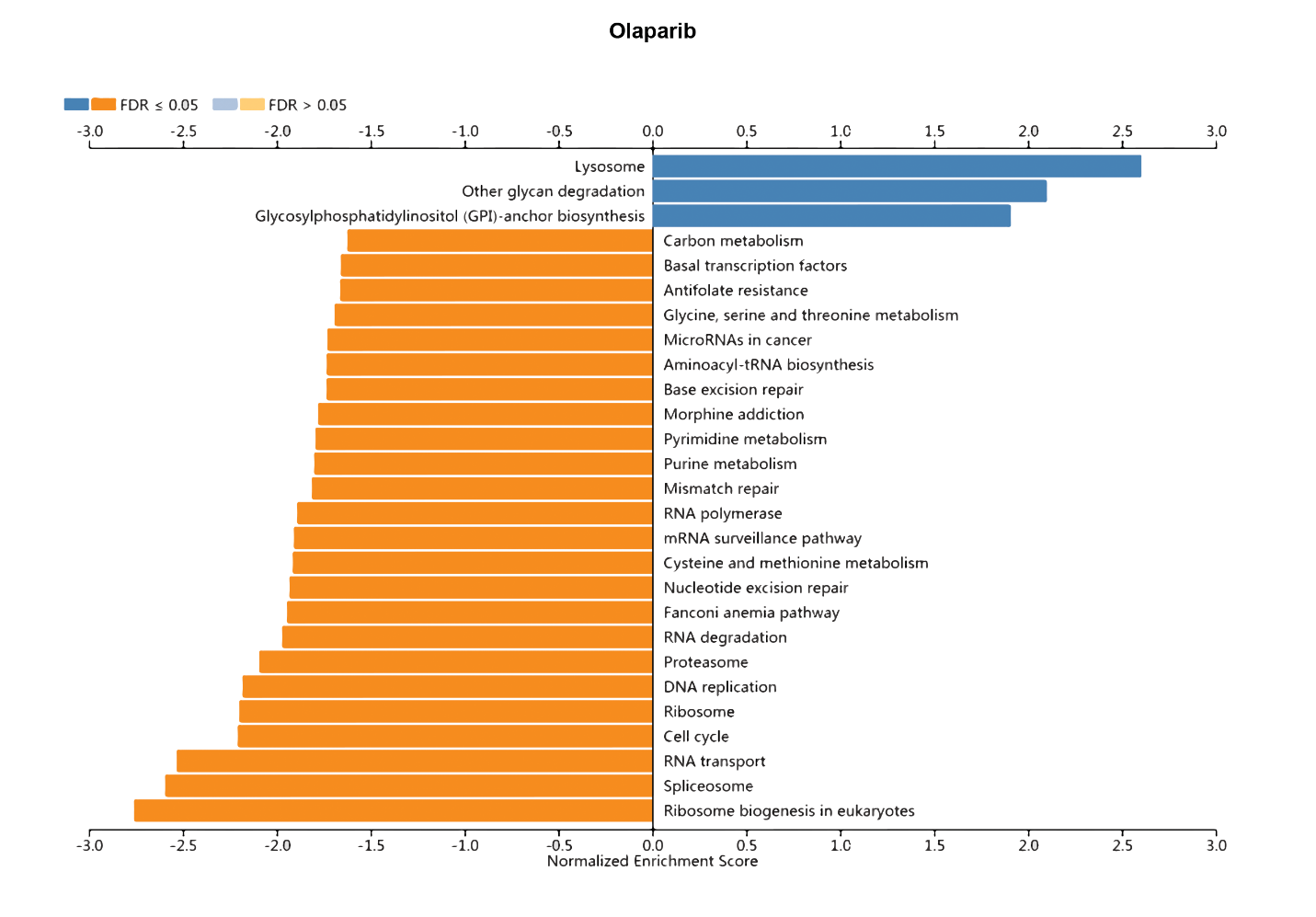


Figure S5.

Summary of gene set enrichment analysis (GSEA) of genes ranked by drug sensitivity-gene expression correlation values using annotated KEGG for olaparib.


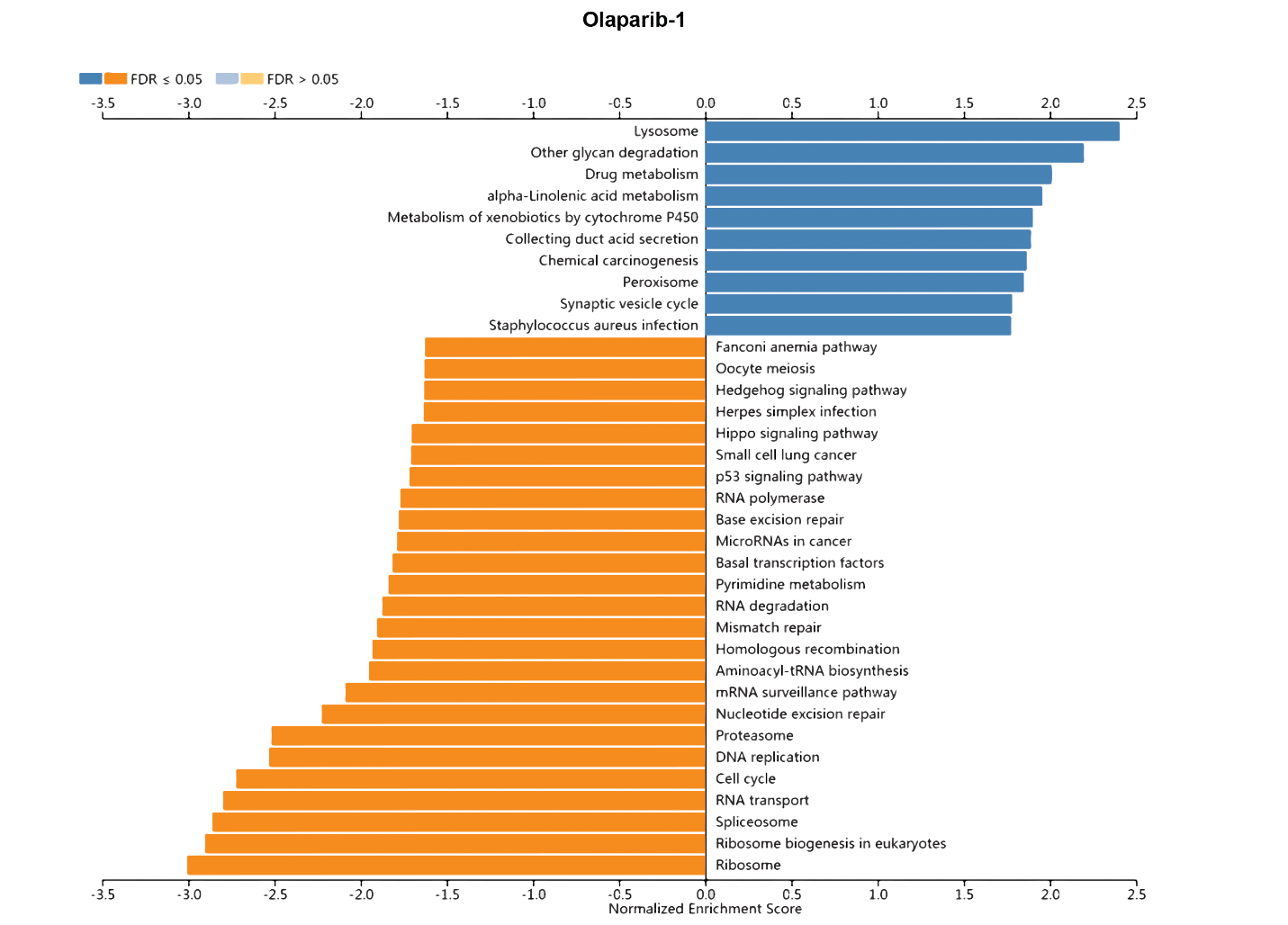


Figure S6.

Summary of gene set enrichment analysis (GSEA) of genes ranked by drug sensitivity-gene expression correlation values using annotated KEGG for olaparib-1.


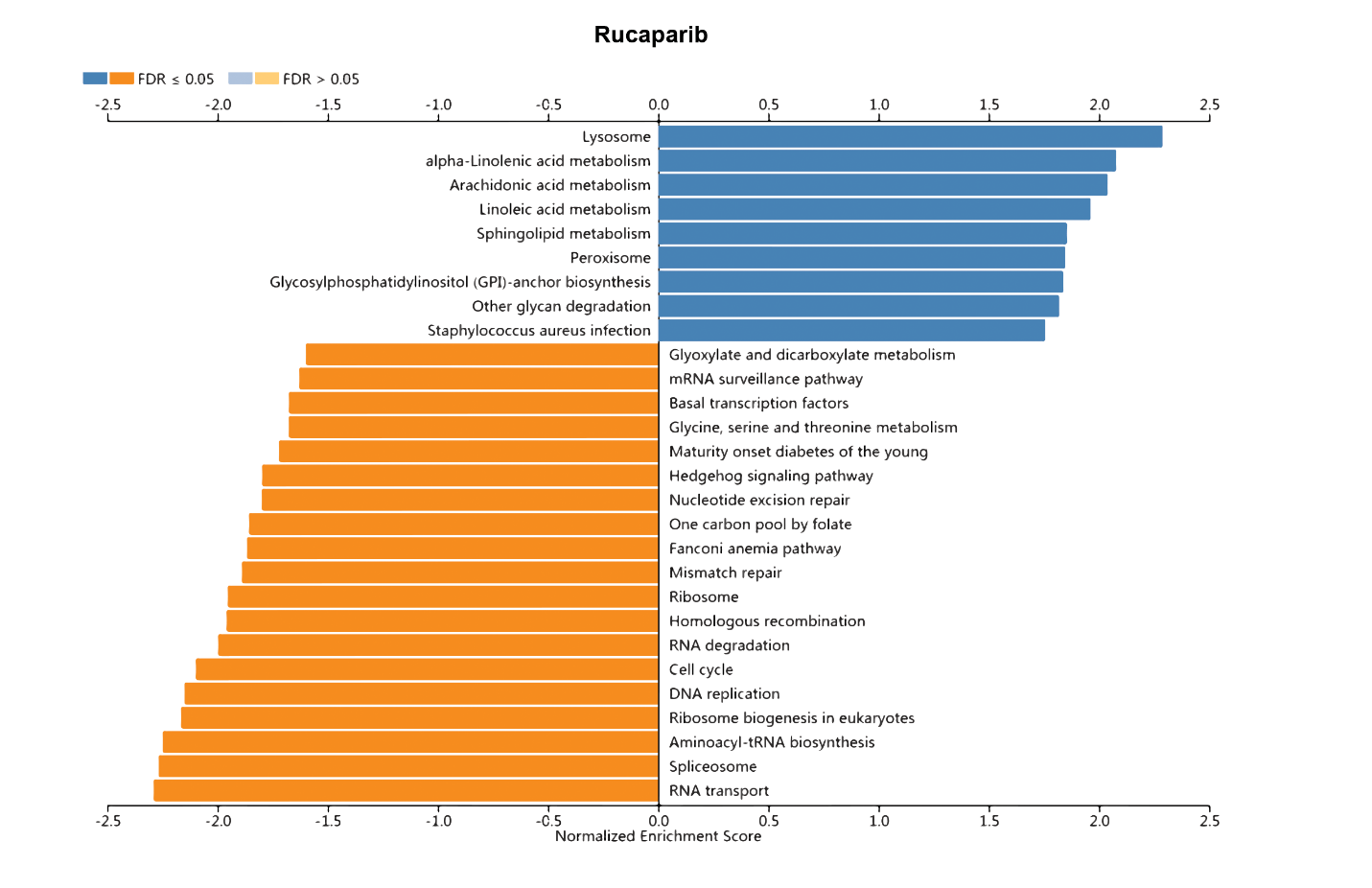


Figure S7.

Summary of gene set enrichment analysis (GSEA) of genes ranked by drug sensitivity-gene expression correlation values using annotated KEGG for rucaparib.


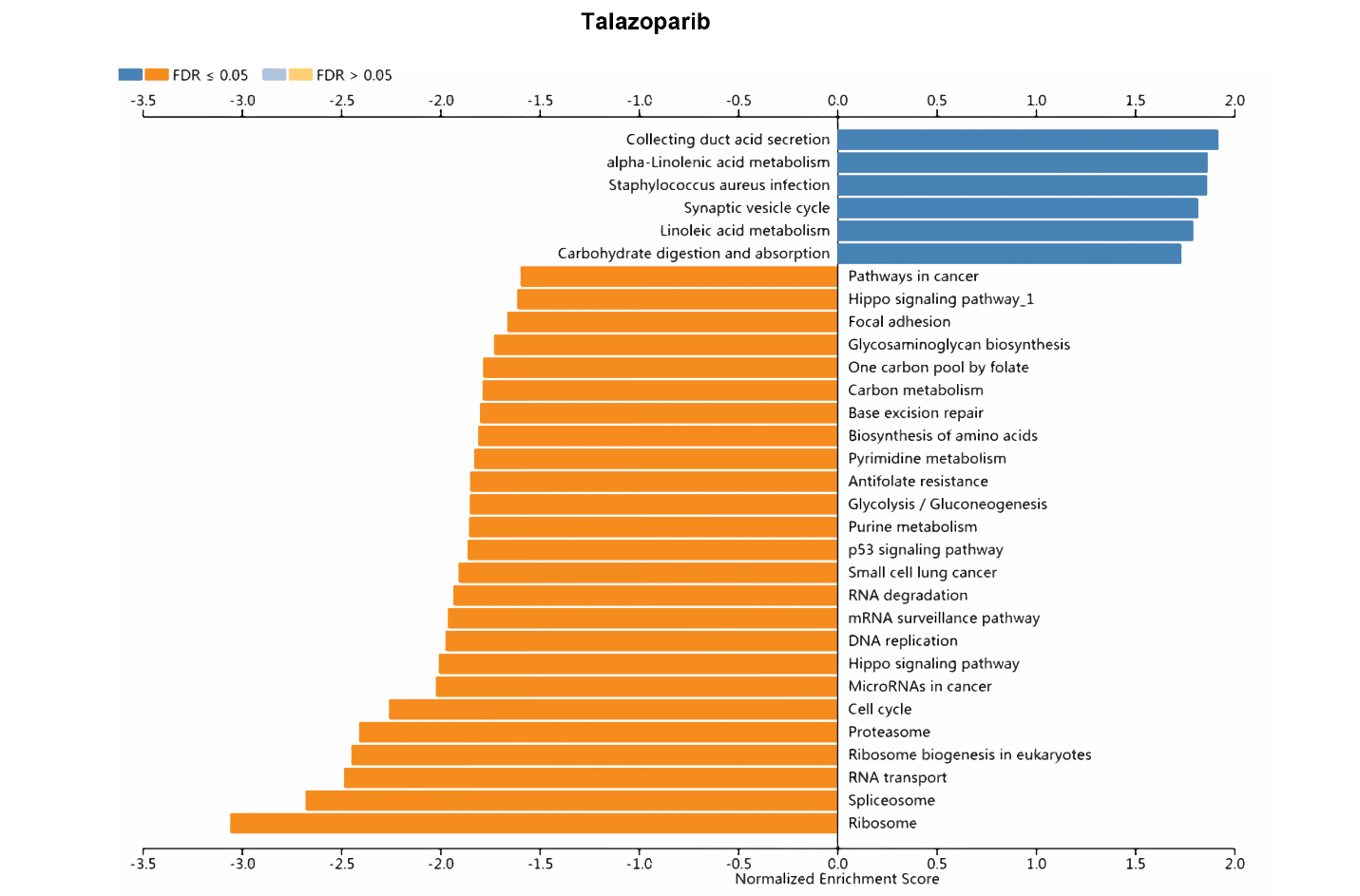


Figure S8.

Summary of gene set enrichment analysis (GSEA) of genes ranked by drug sensitivity-gene expression correlation values using annotated KEGG for talazoparib.


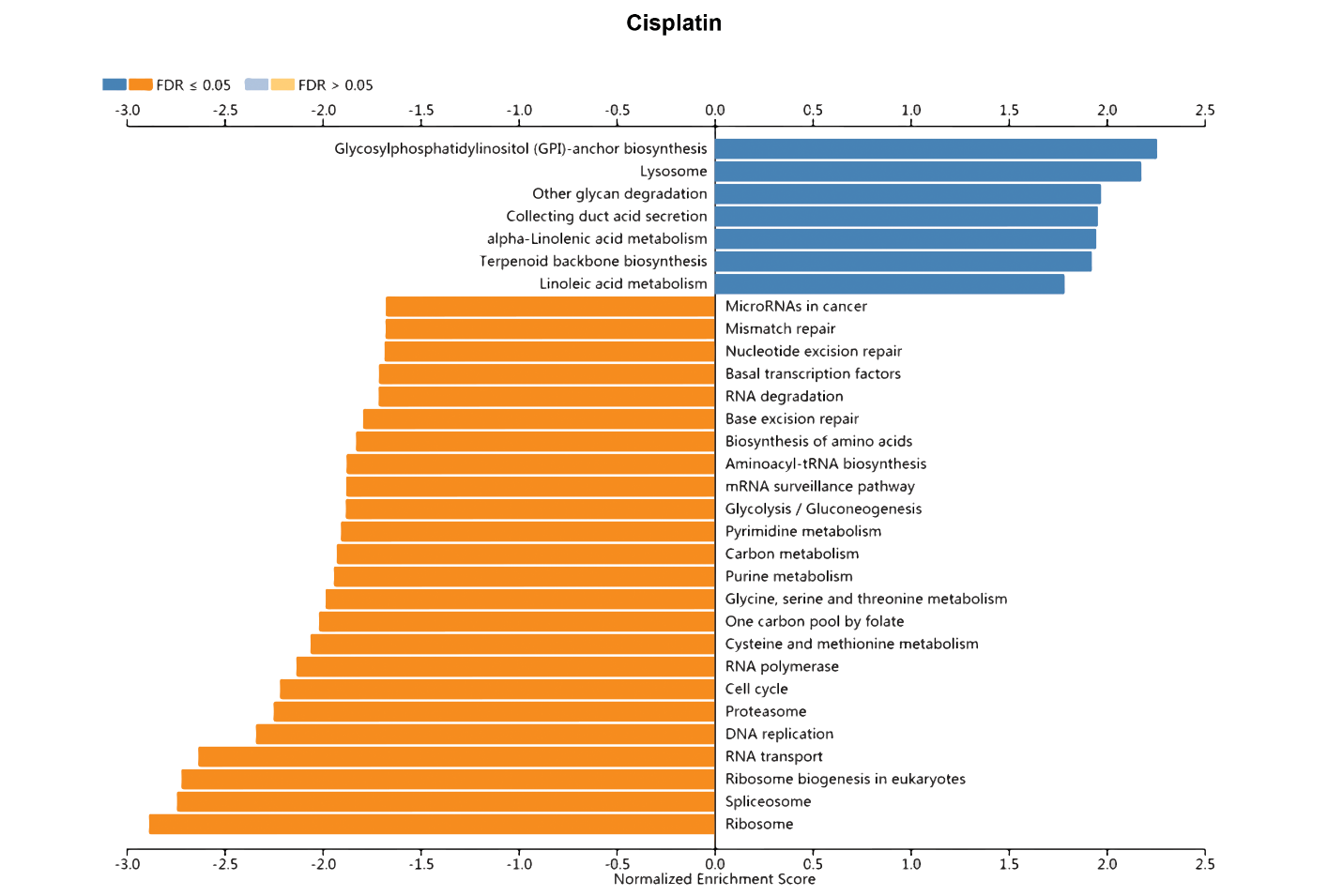


Figure S9.

Summary of gene set enrichment analysis (GSEA) of genes ranked by drug sensitivity-gene expression correlation values using annotated KEGG for cisplatin.


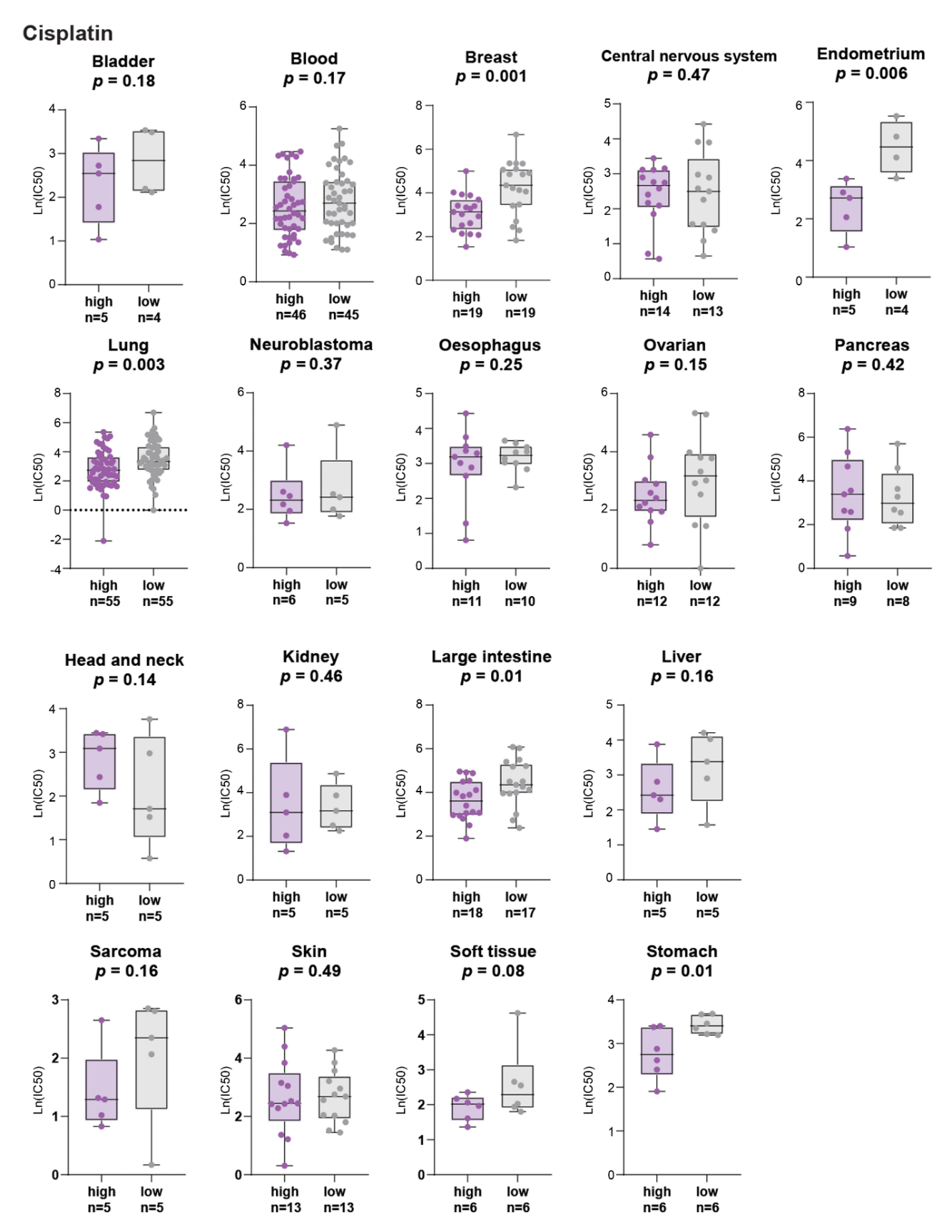


Figure S10.

Prediction of drug response of cell lines to cisplatin via the gene panel. The ln(IC_50_) values for cisplatin in cell lines with high and low expression of the genes in the gene panel from different tumor types. The P values are calculated using one-sided t-tests.


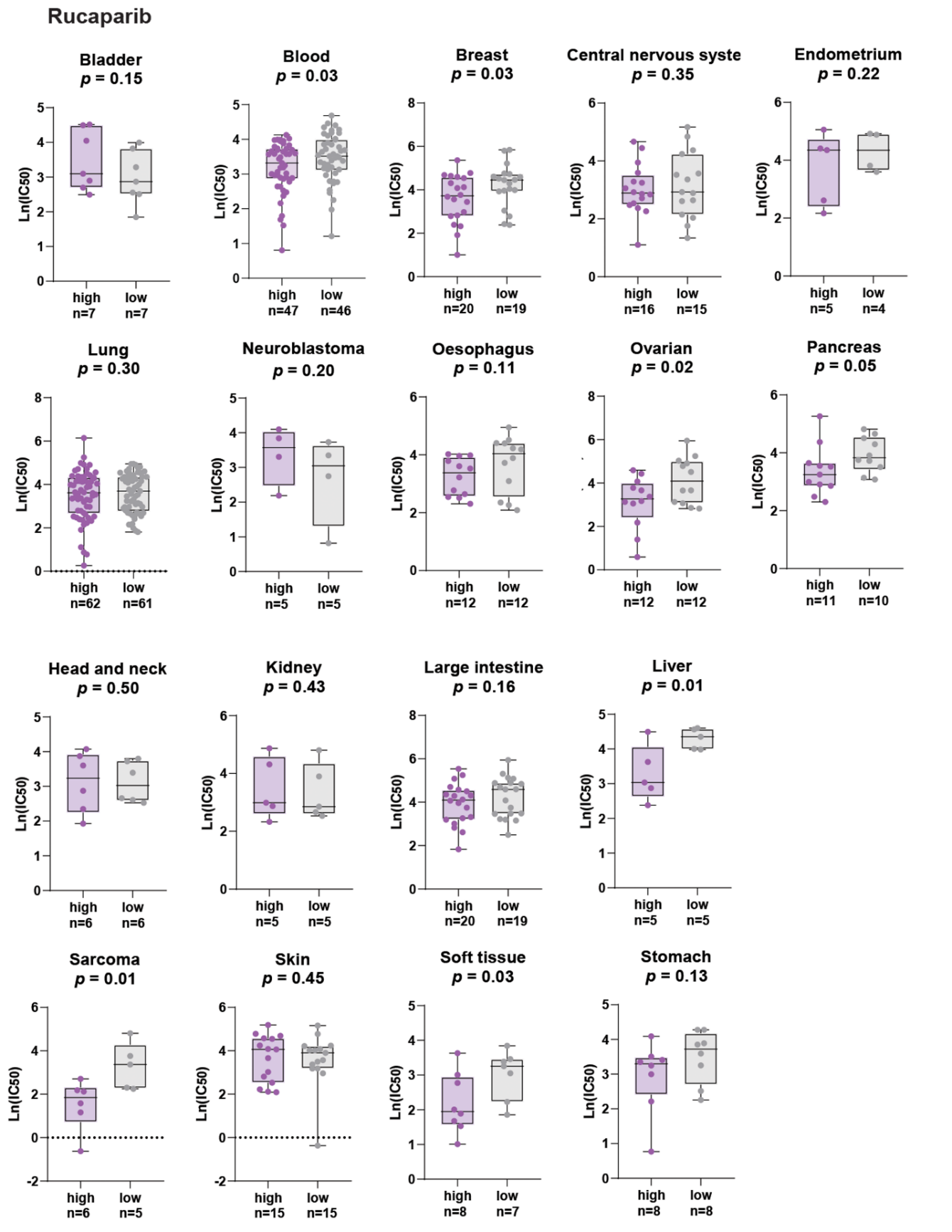


Figure S11.

Prediction of drug response of cell lines to PARP inhibitors via the gene panel. The ln(IC_50_) values for rucaparib in cell lines with high and low expression of the indicated genes in the panel from different tumor types. The P values are calculated using one-sided t-tests.


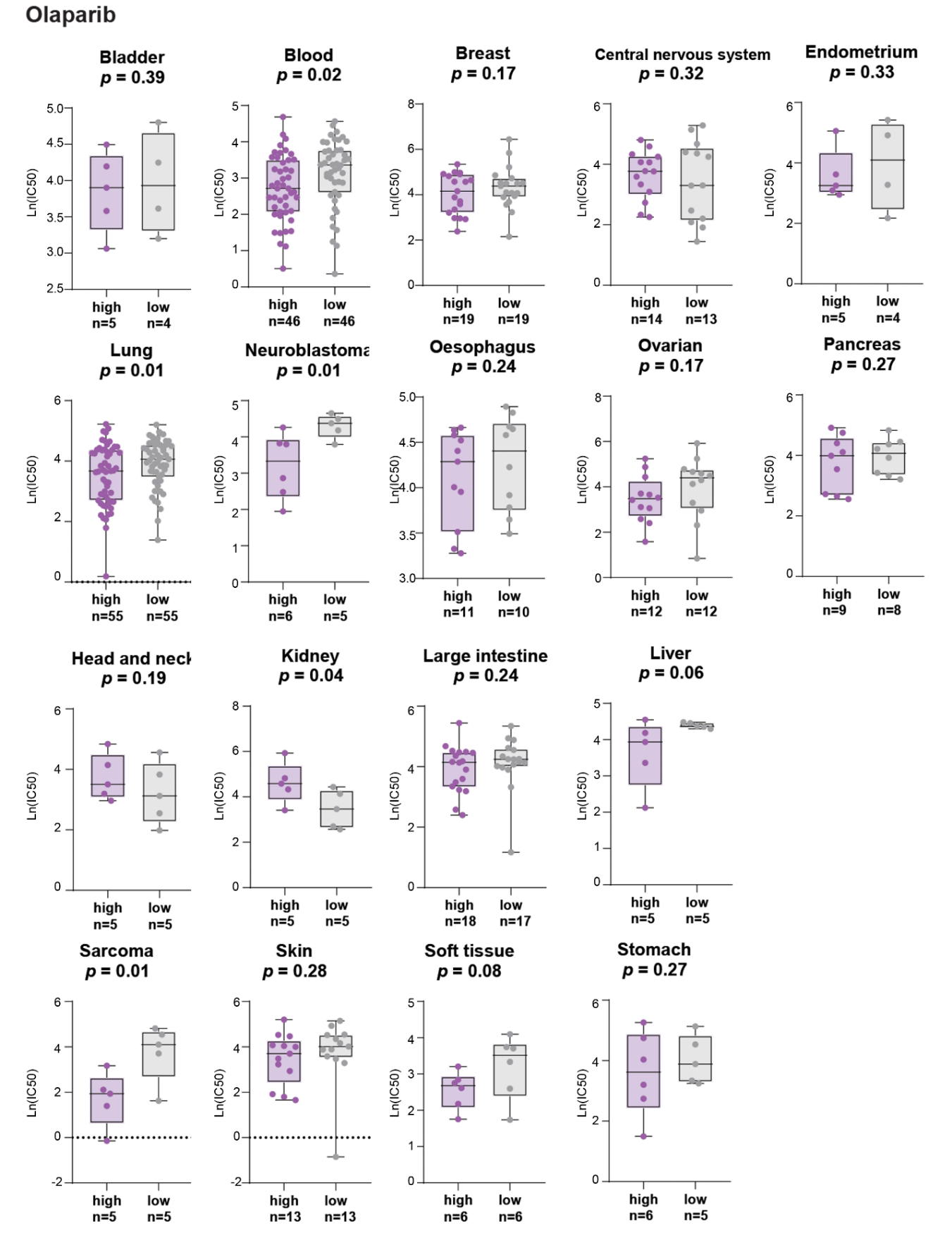


Figure S12.

Prediction of PARP inhibitor response of cell lines via the gene panel. The ln(IC_50_) values for olaparib in cell lines from different tumor types with high and low expression of genes in the gene panel. The P values are calculated using one-sided t-tests.


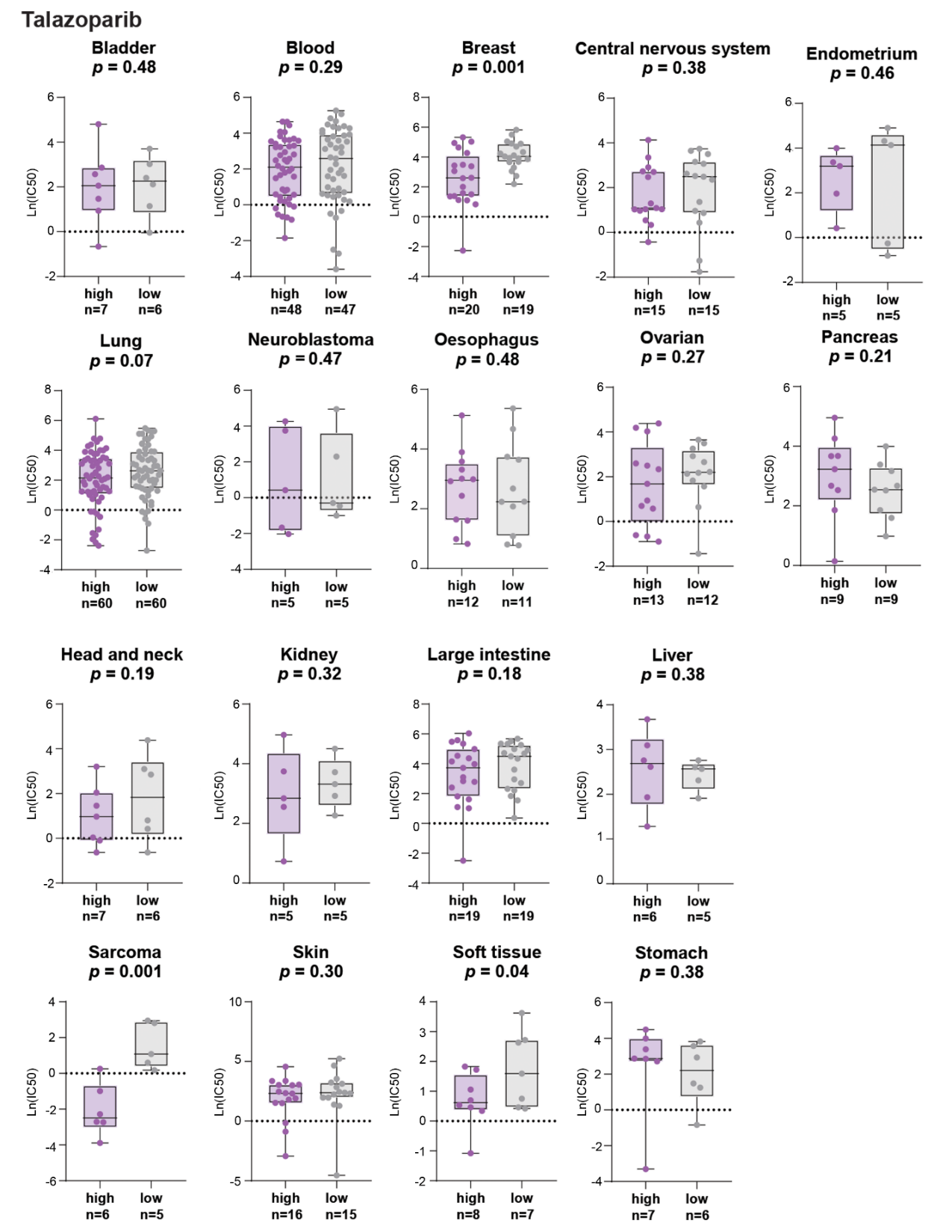


Figure S13.

Prediction of PARP inhibitor response of cell lines via the gene panel. The ln(IC_50_) values for talazoparib in cell lines from different tumor types with high and low expression of genes in the gene panel. The P values are calculated using one-sided t-tests.


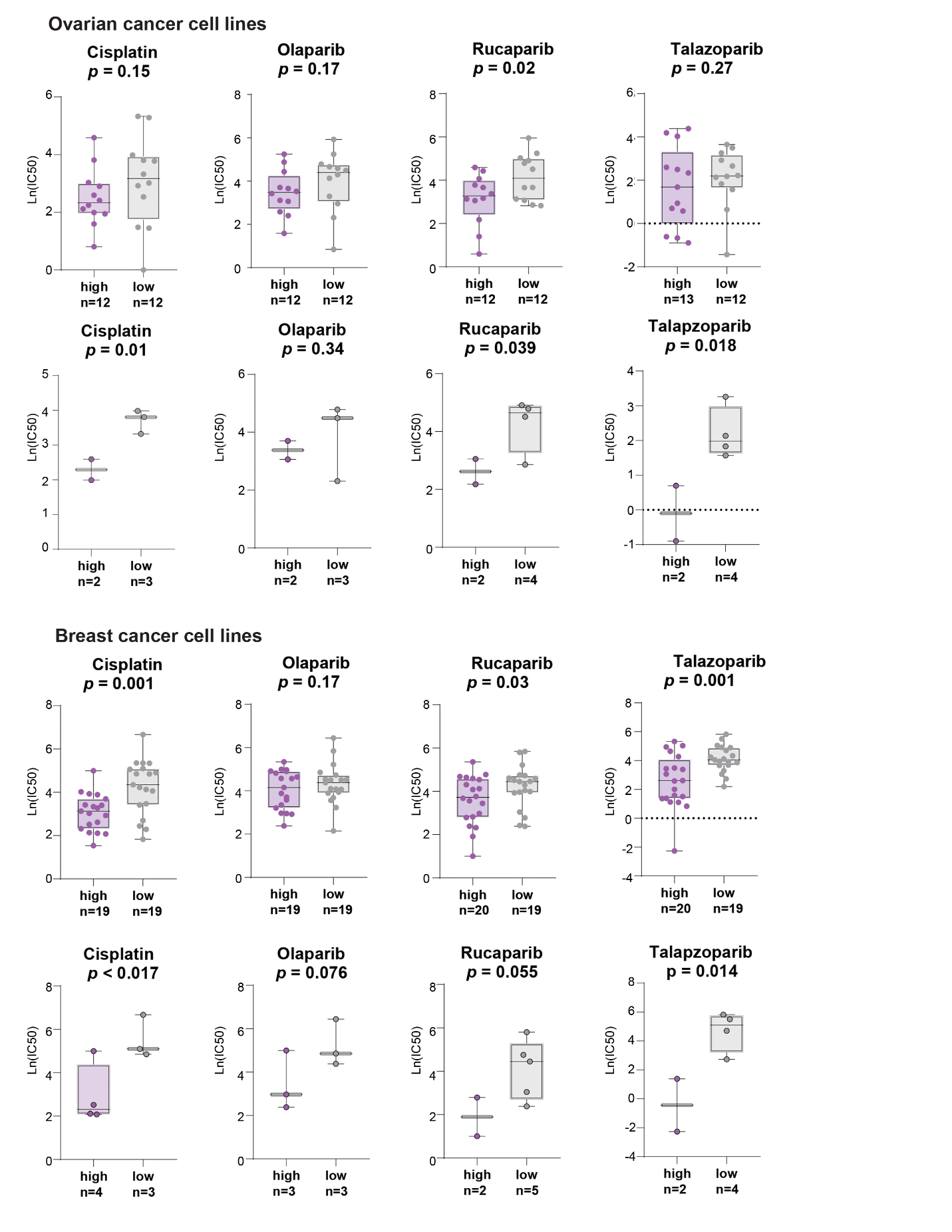


Figure S14.

Prediction of drug response of cell lines to cisplatin and PARP inhibitors via combined examination of both gene panel and HR status. (a) Upper panel: The ln(IC_50_) values of cisplatin, olaparib, rucaparib and talazoparib for ovarian cancer cell lines from the high and low expression groups. Lower panel: the ln(IC_50_) values of cisplatin, olaparib, rucaparib and talazoparib for ovarian cancer cell lines with normal HR function from the high and low expression groups. (b) Upper panel: the ln(IC_50_) values of cisplatin, olaparib, rucaparib and talazoparib for breast cancer cell lines from the high and low expression groups. Lower panel: the ln(IC_50_) values of cisplatin, olaparib, rucaparib and talazoparib for breast cancer cell lines with normal HR function from the high and low expression groups. The P-values are calculated using one-sided t-tests.


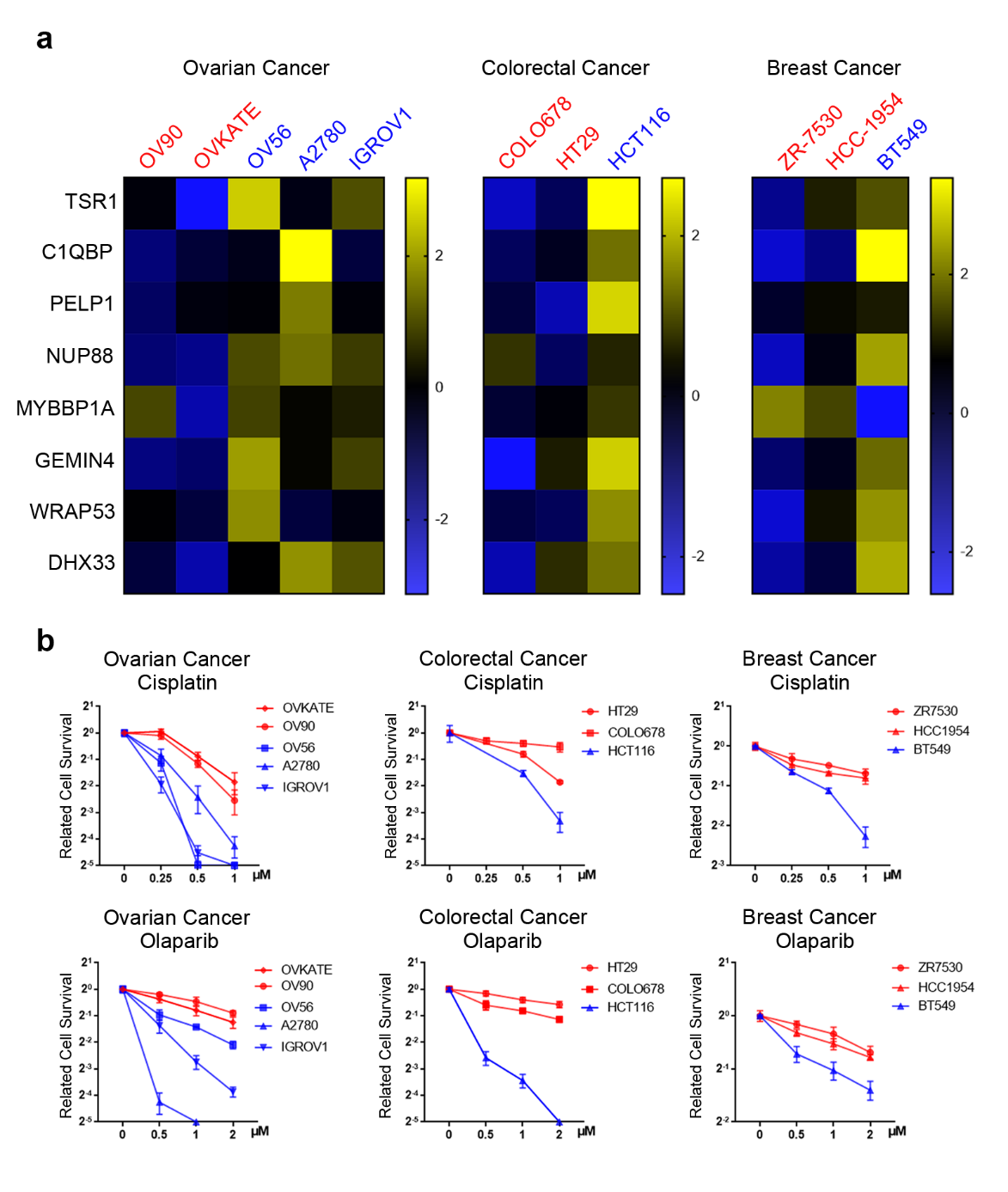


Figure S15.

The high level of genes in this gene panel indicates susceptibility of cell lines to cisplatin/PARP inhibitor. (a) The qPCR results of the biomarkers separated all cell lines into high and low expression groups of the gene panel for each cancer type. (b) The sensitivity to cisplatin or olaparib was assessed by colony formation assay in the indicated cells. Red labels: cell lines with low expression of the gene panel; Blue labels: cell lines with high expression of the gene panel.


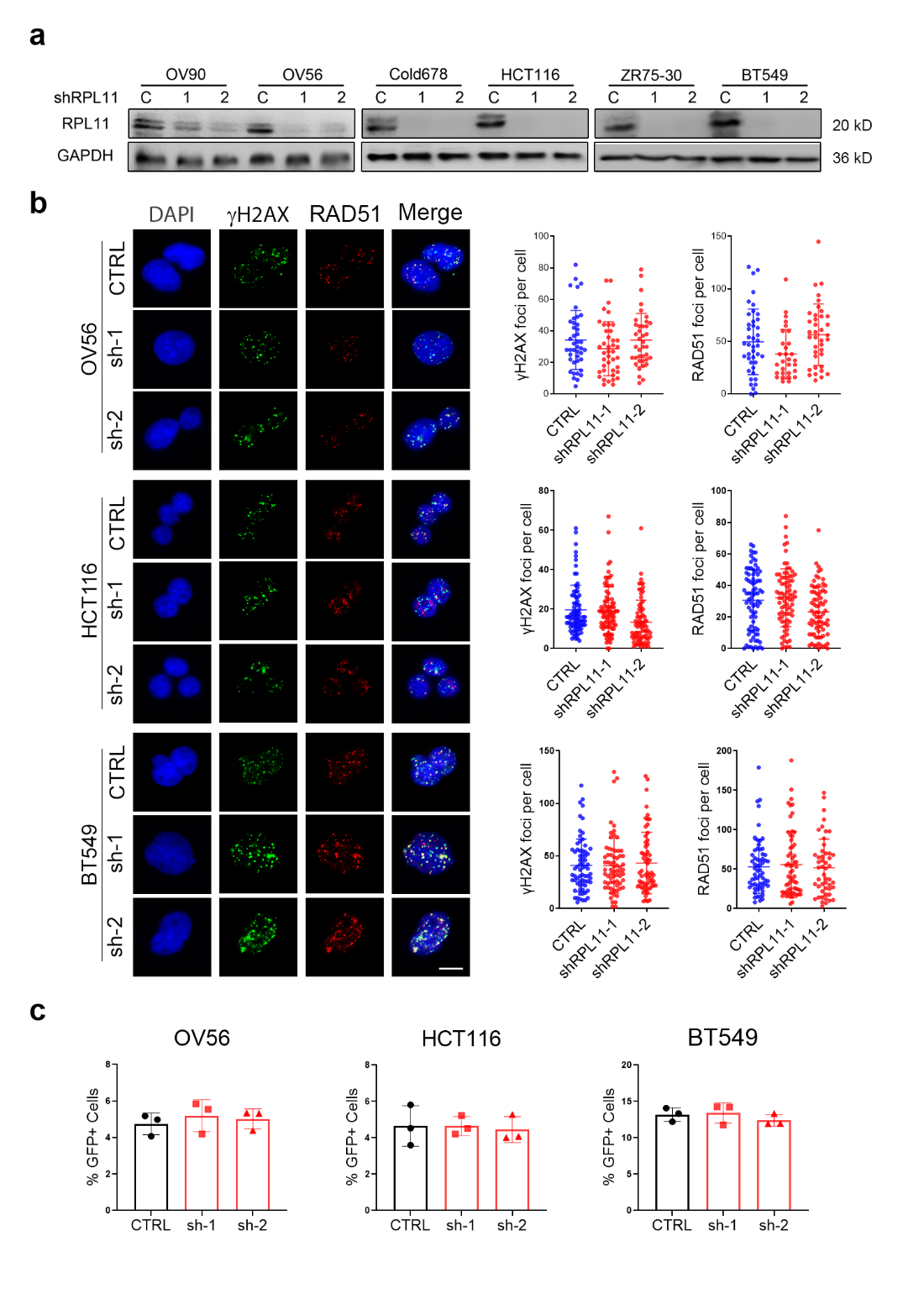


Figure S16.

Knockdown of RPL11 does not affect HR functional status in cells with high expression of the gene panel. (a) The interference efficiency of RPL11 was confirmed by Western Blot. (b) Indicated cells were treated with 2 Gy IR. The γH2AX and RAD51 foci in indicated cell lines were detected and quantified based on immunofluorescent staining (Scale bar: 5 μm). (c) HR efficiency was determined by DNA repair assay.


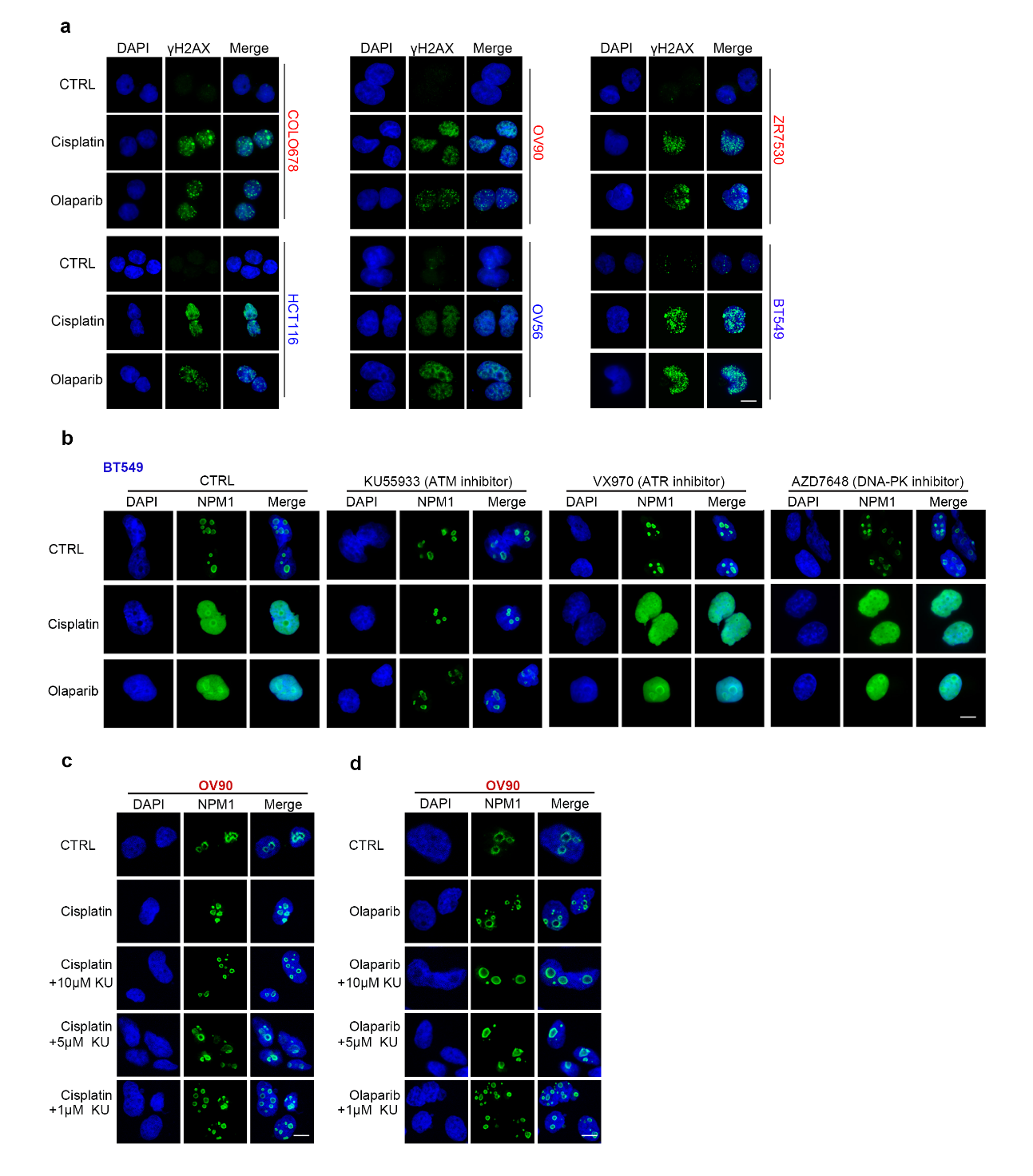


Figure S17.

ATM signaling balances the cell fate by simultaneously controlling both HR repair and ribosomal stress during PARP inhibitor/cisplatin treatment. (a) Indicated cells were treated with cisplatin or olaparib for 6 hours, and then γH2AX foci in indicated cell lines were detected by IF (Scale bar: 5 μm). (b) BT549 cells were pre-treated with KU55933(ATM inhibitor), VX970(ATR inhibitor) or AZD7648(DNA-PK inhibitor) for 1 hour, the localization of NPM1 was detected by immunofluorescence to indicate the ribosomal stress induced by cisplatin or Olaparib (Scale bar: 5 μm). (c-d) OV90 cells were pre-treated with indicated concentrations of KU55933 for 1 hour. The nucleolar localization of NPM1 was detected to indicated the ribosomal stress level caused by cisplatin or Olaparib (Scale bar: 5 μm). Red font: low expression of the gene panel cell lines. Blue front: high expression of the gene panel cell lines.


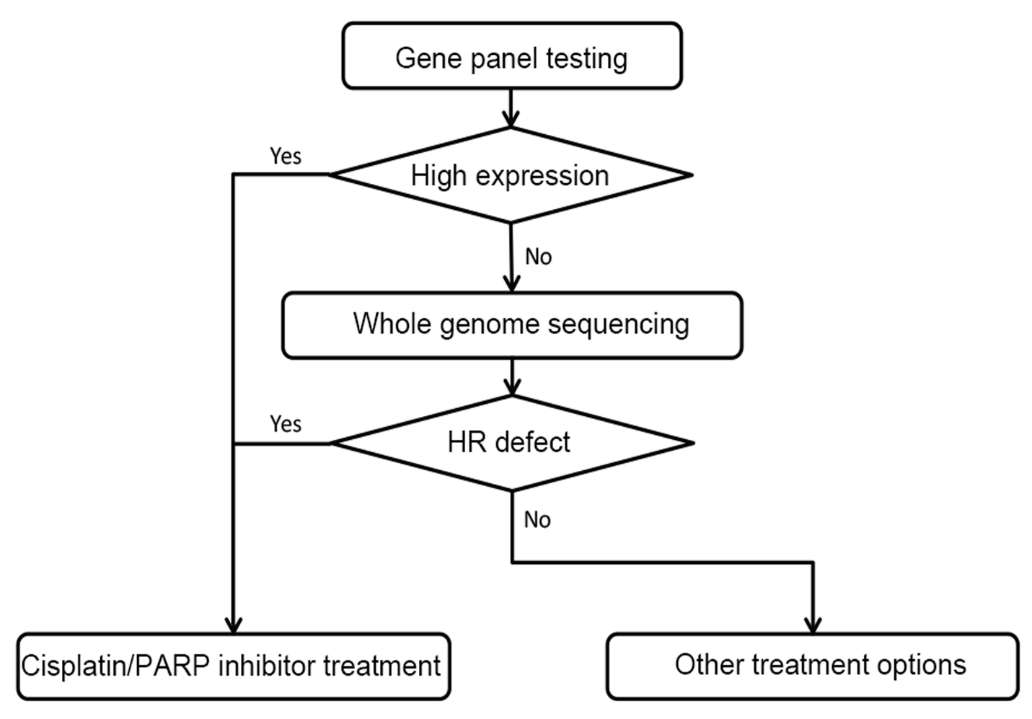


Figure S18.

The extended principle for the usage of PARP inhibitors and cisplatin. Patients with high expression of these biomarkers can be screened out to receive PARP inhibitor/cisplatin therapy; and the remining patients need to further check for their HR status by the current clinical methods. We suggest checking the gene panel first would be faster and more cost effective, and it can exempt many patients from expensive HR status detection.


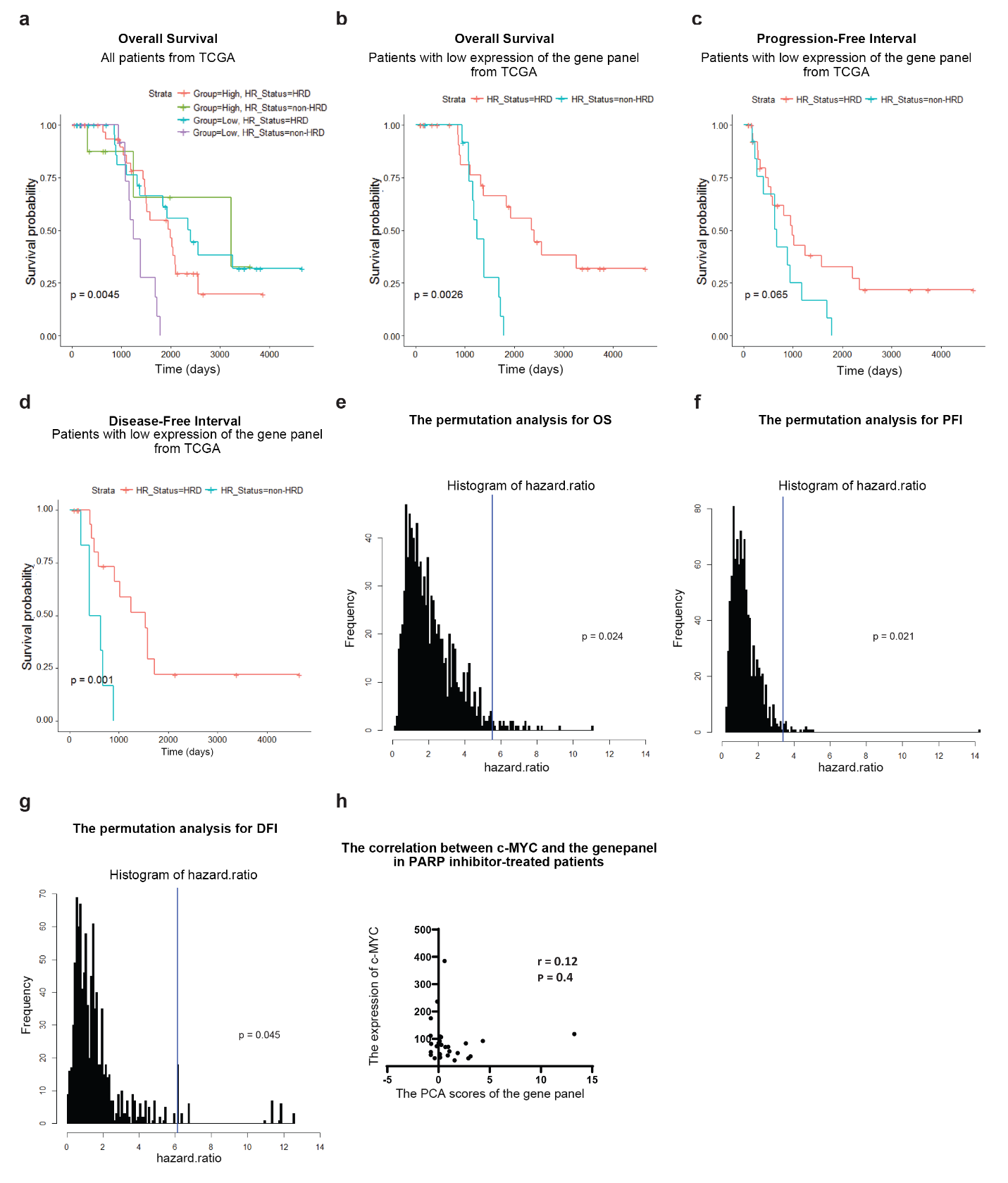


Figure S19.

The combined examination of gene panel and HR status effectively predicts clinical cancer response to PARP inhibitor and cisplatin. (a) The two-factor overall survival analysis the gene panel and HR status for cisplatin-treated patients from TCGA (n=84). (b) The overall survival analysis via univariate Cox regression with HR status for cisplatin-treated patients with low expression of the gene panel from TCGA (hazard ratio=4.082; 95%CI 1.529-10.9; p = 0.0026; n=42). (c) The progression-free survival analysis with HR status for cisplatin-treated patients with low expression of the gene panel from TCGA (hazard ratio=2.031; 95%CI 0.9434-4.373; p = 0.065; n=42). (d) The disease-free survival analysis with HR status for cisplatin-treated patients with low expression of the gene panel (hazard ratio=6.551; 95%CI 1.813-23.67; p = 0.001; n=24). (e-g) The permutation test (1000 times) to compare the performance of randomly selected gene lists from the top genes negatively correlated to PARP inhibitor/cisplatin sensitivity with our gene panel to predict the overall survival (p=0.024), progression-free interval (p=0.021) and disease-free interval (p=0.045) of cisplatin-treated patients with normal HR function. One-tailed test. (h) Correlation between the transcriptional expression levels of c-MYC and the PCA scores of the gene panel in PARP inhibitor-treated patients (r=0.12; p=0.4).


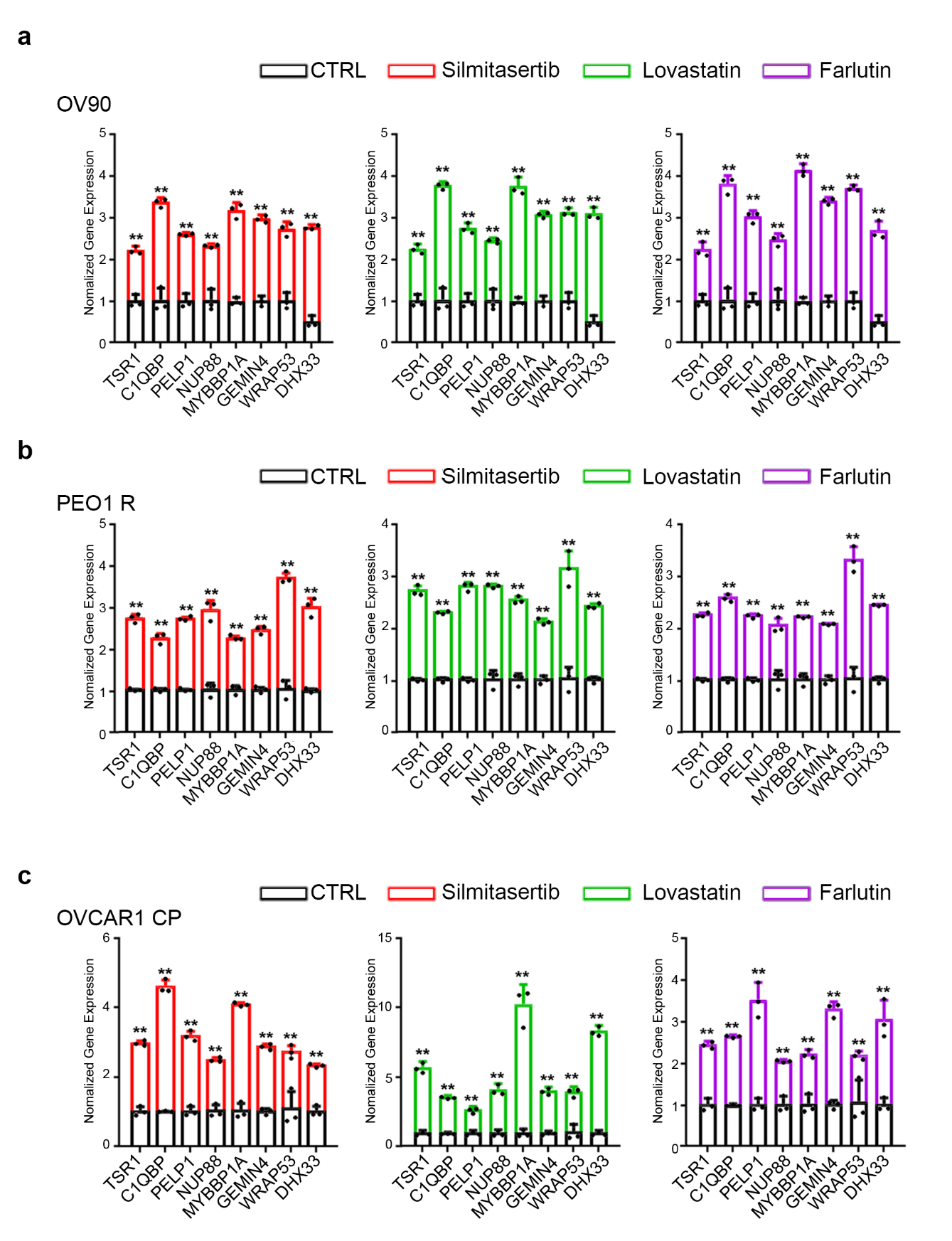


Figure S20.

The effects of the three marketed drugs on PARP inhibitor/cisplatin resistant cell lines. (a-c) OV90 (a), PEO1 R (b) and OVCAR1 CP (c) cells were pre-treated with the three listed drugs for 24 hours. The expression levels of the 8 genes were detected by qRT-PCR. ** *p*<0.01, ns not significant (compared with CTRL).


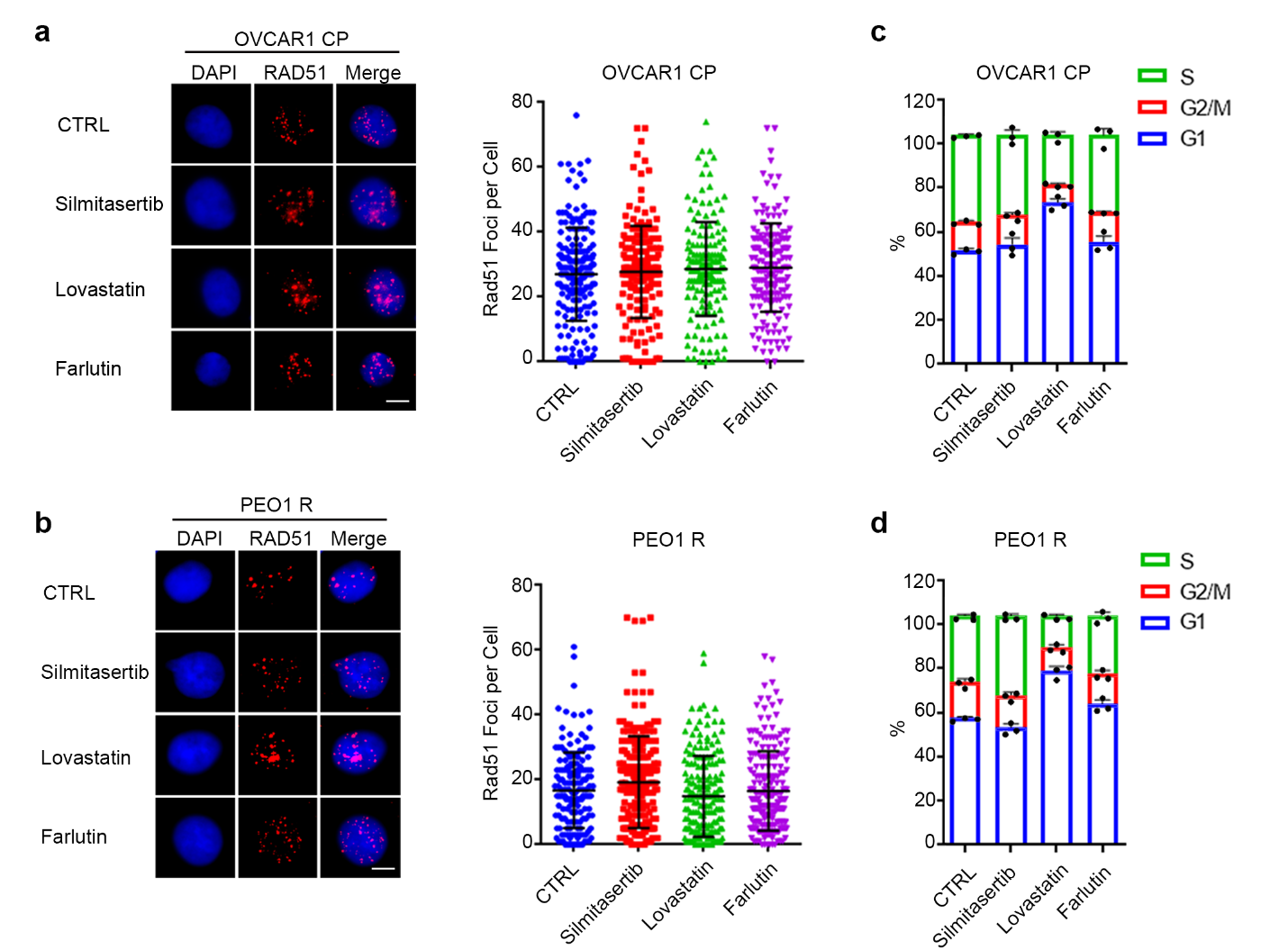


Figure S21.

The effects of the three marketed drugs on PARP inhibitor/cisplatin resistant cell lines. (a-b) OVCAR1 CP (a) and PEO1 R (b) cells were pre-treated with the three listed drugs for 24 hours, then treated with 2 Gy IR (Scale bar: 5 μm). RAD51 foci were detected by immunofluorescent staining. (c-d) OVCAR1 CP (c) and PEO1 R (d) cells were pre-treated with the three listed drugs for 24 hours, then cell cycle analysis was performed by flow cytometry.


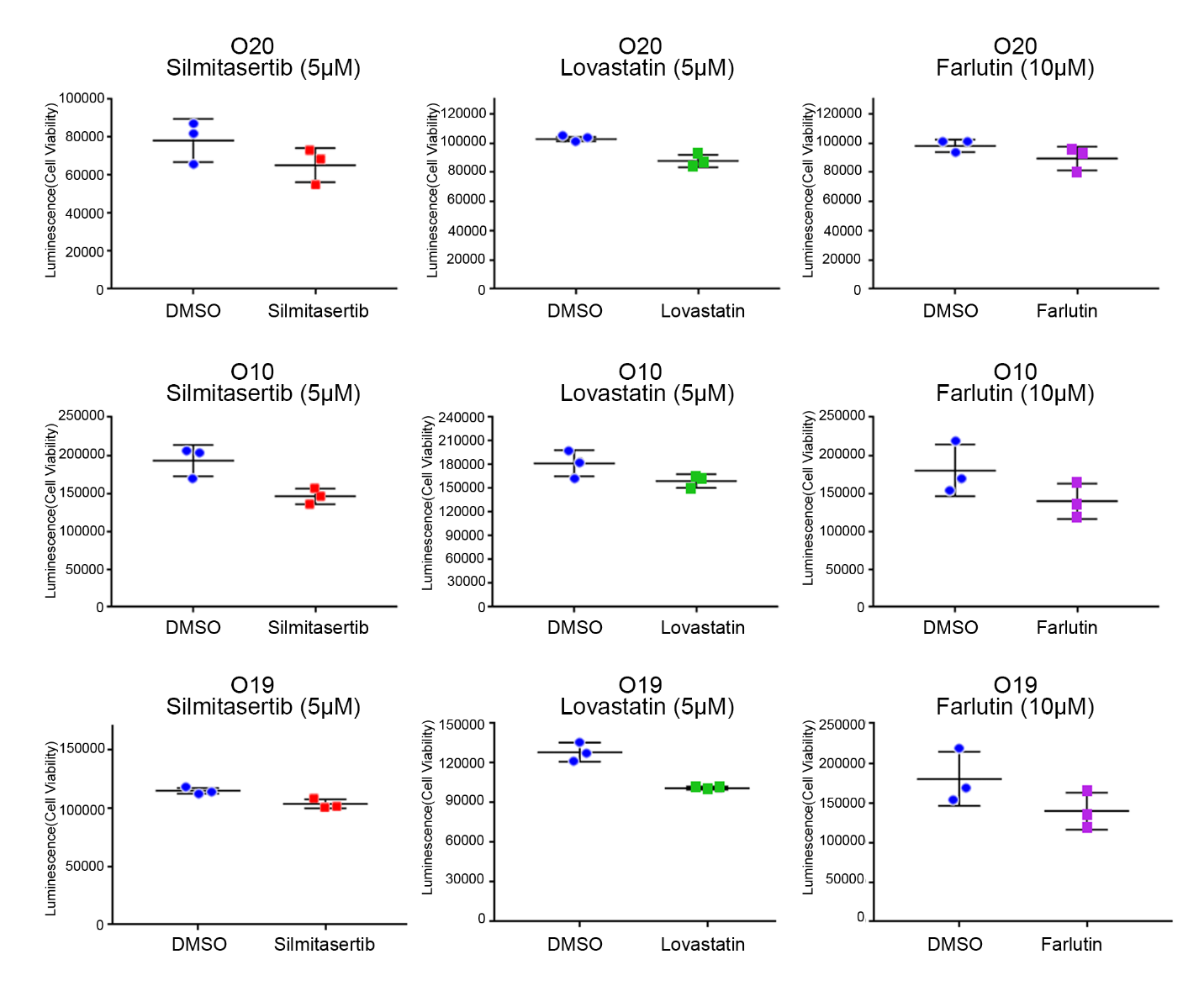


Figure S22.

The effects of the synergistic lethal dose of these 3 listed drugs with cisplatin/PARP inhibitor on these original resistant organoid models were assessed by cell viability assay, separately.

Table S1.

Drug information.

| **Drug name** | **Putative target** |
| --- | --- |
| cisplatin | DNA crosslinder |
| olaparib | PARP1/2 inhibitor |
| rucaparib | PARP1/2 inhibitor |
| talazoparib | PARP1/2 inhibitor |
| temozolomide | DNA alkylating agent |
| Etoposide | TOP2 inhibitor |
| 5-FU | Antimetabolite (DNA & RNA) |
| JQ12 | HDAC1/2 inhibitor |
| Mitomycin-C | DNA crosslinder |
| Bleomycin | dsDNA break induction |

Table S2.

The signature module for each drug derived by WGCNA analysis.

Table S3.

Pearson correlation analysis between basal gene expression and drug sensitivity of PARP inhibitors and cisplatin.

Table S4.

The 21 candidate genes derived by taking the intersection of genes involved in the drug signature module and the top genes negatively correlated with sensitivity to these drugs (FDR < 0.05).

| Seq No | Gene name | Seq No | Gene name | Seq No | Gene name |
| --- | --- | --- | --- | --- | --- |
| 1 | MYBBP1A | 8 | PFAS | 15 | FXR2 |
| 2 | UBE2G1 | 9 | SCO1 | 16 | TIMM22 |
| 3 | EIF4A1 | 10 | YWHAE | 17 | RPA1 |
| 4 | NUP88 | 11 | RNMTL1 | 18 | METTL16 |
| 5 | GEMIN4 | 12 | DHX33 | 19 | PRPF8 |
| 6 | PELP1 | 13 | WRAP53 | 20 | CRK |
| 7 | C1QBP | 14 | TSR1 | 21 | ANKFY1 |

Table S5.

The prediction on response of ovarian cancer cell lines to PARP inhibitors and cisplatin.

Table S6.

The prediction on response of breast cancer cell lines to PARP inhibitors and cisplatin.

Table S7.

The prediction on response of ovarian cancer organoids to PARP inhibitor and cisplatin.

|  | **Overall rank of the gene panel** | | **Olaparib(IC50)** | **Response to**  **olaparib** | **Cisplatin(IC50)** | **Response to cisplatin** | **Note** |
| --- | --- | --- | --- | --- | --- | --- | --- |
| O1 | 9.138483956 | | 73.39 | S | 0.9666 | S |  |
| O2 | 1.58978673 | | 45.32 | S | 8.898 | S |  |
| O3 | 0.846251937 | | 55.65 | S | 22.77 | S |  |
| O4 | 0.165870797 | | 88.5 | S | 18.96 | S |  |
| O5 | 0.125748611 | | 44.05 | S | 24.41 | S |  |
| O6 | -0.086867125 | | 4.964 | S | 14.53 | S |  |
| O7 | -0.09400353 | | 205.4 | R | 22.93 | S |  |
| O8 | -0.289327247 | | 109 | R | 43.82 | R |  |
| O9 | -0.303934144 | | 9.509 | S | 25.76 | S | FANCI: exon3 c.97C>T p.L33F; missense mutation. BRIP1: exon8 c.1139G>A p.S380N; missense mutation. |
| O10 | -0.436521493 | | 191.4 | R | 43.95 | R |  |
| O11 | -0.459480037 | | 74.47 | S | 10.87 | S | ATRX: exon9 c.2984C>T p.P995L; missense mutation. |
| O12 | -0.569497867 | | 89.39 | S | 15.57 | S | FANCD2: exon3 c.141C>G p.I47M; missense mutation. ATM: exon11 c.1744T>C p.F582L; missense mutation. BRCA2: exon27 c.9976A>T p.K3326; nonsense mutation. ATRX: exon21 c.5424T>G p.Y1808; nonsense mutation. |
| O13 | -0.607105054 | | 95.11 | R | 31.35 | R |  |
| O14 | -0.609827653 | | 160.4 | R | 30.07 | R |  |
| O15 | -0.818377087 | | 197.3 | R | 56.09 | R |  |
| O16 | -1.036624051 | | 103 | R | 81.28 | R |  |
| O17 | -1.412408313 | | 255 | R | 29.7 | R |  |
| O18 | -1.471137891 | | 63.83 | S | 17.89 | S | SMAD3: exon8 c.1081G>A p.E361K; missense mutation. |
| O19 | -1.723827434 | | 154 | R | 41.56 | R |  |
| O20 | -1.947203104 | 165.2 | | R | 43.95 | R |  |

Table S8.

The mutation information of ovarian cancer organoids derived from 600 gene panel detection.

| Model (ID) | Gene | Alteration site | Alteration type | Fractional Abundance |
| --- | --- | --- | --- | --- |
| O9 | PRCC | exon3 c.602C>G p.P201R | Missense mutation; | 44.10% |
|  | BRAF | intron6 c.861-421A>T | Intron variation | 53.33% |
|  | ADGRA2 | exon19 c.3573C>A p.R1191= | Synonymous mutation | 53.25% |
|  | SYK | exon2 c.23A>T p.D8V | Missense mutation; | 46.76% |
|  | NCOR2 | exon16 c.1529_1531dup p.Q510dup | in-frame insertion | 52.46% |
|  | FANCI | exon3 c.97C>T p.L33F | Missense mutation; | 46.04% |
|  | BRIP1 | exon8 c.1139G>A p.S380N | Missense mutation; | 47.19% |
|  | PPP2R1A | exon13 c.1546A>G p.I516V | Missense mutation; | 48.55% |
|  | RUNX1 | exon9 c.1270T>C p.S424P | Missense mutation; | 7.63% |
| O11 | CBLB | exon14 c.2078C>G p.S693C | Missense mutation; | 44.26% |
|  | TERT | exon4 c.1787T>G p.V596G | Missense mutation; | 47.80% |
|  | MET | intron14 c.3029-721del | Intron alteration | 2.91% |
|  | PTPRD | exon27 c.2422G>C p.D808H | Missense mutation; | 98.84% |
|  | NOTCH1 | exon34 c.6546G>T p.K2182N | Missense mutation; | 33.33% |
|  | HNF1A | exon1 c.29C>T p.T10M | Missense mutation; | 99.71% |
|  | MYH7 | exon14 c.1388C>T p.A463V | Missense mutation; | 98.95% |
|  | MGA | exon15 c.5098G>A p.V1700M | Missense mutation; | 48.03% |
|  | TP53 | exon10 c.1025G>C p.R342P | Missense mutation; | 97.90% |
|  | ERBB2 | intron16 c.1946+561C>G | Intron alteration | 100.00% |
|  | AR | intron2 c.1769-61G>A | Intron alteration | 57.14% |
|  | ATRX | exon9 c.2984C>T p.P995L | Missense mutation; | 22.49% |
| O18 | EOMES | exon1 c.378_380del p.A130del | in-frame deletion | 5.26% |
|  | PIK3R1 | exon13 c.1712T>A p.I571N | Missense mutation; | 16.26% |
|  | ZNF703 | exon2 c.698A>G p.N233S | Missense mutation; | 46.90% |
|  | EMSY | exon8 c.892C>T p.H298Y | Missense mutation; | 46.72% |
|  | KMT2A | exon3 c.2233C>T p.R745* | nonsense mutation | 12.76% |
|  | KRAS | exon2 c.35G>A p.G12D | Missense mutation; | 9.98% |
|  | NCOR2 | exon16 c.1529_1531dup p.Q510dup | in-frame insersion | 65.00% |
|  | SMAD3 | exon8 c.1081G>A p.E361K | Missense mutation; | 14.92% |
|  | SMAD4 | exon10 c.1156G>A p.G386S | Missense mutation; | 13.71% |
|  | AMER1 | exon2 c.2434G>A p.A812T | Missense mutation; | 46.76% |
| O12 | PIK3CD | exon22 c.2722T>G p.F908V | Missense mutation; | 5.11% |
|  | ELF3 | exon2 c.15T>G p.C5W | Missense mutation; | 8.91% |
|  | ASXL2 | exon12 c.1243T>C p.S415P | Missense mutation; | 46.12% |
|  | MSH6 | exon4 c.3055A>C p.I1019L | Missense mutation; | 5.85% |
|  | IRS1 | exon1 c.1706C>T p.P569L | Missense mutation; | 51.15% |
|  | UGT1A1 | exon1 c.686C>T p.P229L | Missense mutation; | 50.51% |
|  | FANCD2 | exon3 c.141C>G p.I47M | Missense mutation; | 45.85% |
|  | CARD11 | exon19 c.2542C>T p.R848C | Missense mutation; | 49.29% |
|  | NOTCH1 | exon13 c.2206G>A p.G736R | Missense mutation; | 48.67% |
|  | ATM | exon11 c.1744T>C p.F582L | Missense mutation; | 47.97% |
|  | PIK3C2G | exon28 c.3784C>T p.P1262S | Missense mutation; | 40.87% |
|  | KMT2D | exon39 c.10774A>C p.M3592L | Missense mutation; | 9.86% |
|  | CDK4 | exon7 c.806C>T p.A269V | Missense mutation; | 49.10% |
|  | NCOR2 | exon16 c.1529_1531dup p.Q510dup | in-frame insersion | 8.20% |
|  | NCOR2 | exon16 c.1526_1531dup p.Q509_Q510dup | in-frame insersion | 55.91% |
|  | LATS2 | exon3 c.461A>G p.K154R | Missense mutation; | 50.91% |
|  | BRCA2 | exon27 c.9976A>T p.K3326* | nonsense mutation | 48.77% |
|  | NOTCH3 | exon4 c.532C>T p.P178S | Missense mutation; | 50.80% |
|  | PVR | exon2 c.368G>T p.C123F | Missense mutation; | 50.28% |
|  | RUNX1 | exon9 c.1270T>G p.S424A | Missense mutation; | 26.38% |
|  | RUNX1 | exon9 c.1265A>G p.E422G | Missense mutation; | 20.00% |
|  | AR | exon1 c.1412_1420del p.G471_G473del | in-frame deletion | 59.79% |
|  | ATRX | exon21 c.5424T>G p.Y1808* | nonsense mutation | 3.69% |

Table S9.

The prediction on response of a panel of primary breast cancer PDTX-derived tumor cells (PDTCs) to PARP inhibitor.

Table S10.

The prediction on response of a panel of primary ovarian cancer PDTCs to PARP inhibitor.

Table S11.

The prediction on response of patients with platinum-sensitive relapsed ovarian cancer treated with PARP inhibitor maintenance monotherapy.

**Table S12**

RT-qPCR primer and pLenti-shRNA guide sequences.

| Genes | Primer sequence (5’-3’) | Genes | shRNA guide sequences (5’-3’) |
| --- | --- | --- | --- |
| MYBBP1a | Forward: TGGTGAGCATTGTGGACAG  Reverse: CAAAGGGAAGGAGAACGGG | RPL11 | 1. 1. CATCCGCAAACTCTGTCTCAA   2. CCGCAAACTCTGTCTCAACAT |
| GEMIN4 | Forward: CCAAACCCTGTTGCAGAAG  Reverse: ACAGACCTGCCATGTTGAAG | NUP88 | 1. GCTGCATGGTATCCAAGTGAA  2. CGGCTGAAGATAACTATGGTT |
| PELP1 | Forward: CTTGTATGACCTATTTCCCTCGG  Reverse: GCATCCACCCTAGACAGAAAA | PELP1 | 1. GAAGAGTTTGAGGAAGAATTT  2. GAAGAAGGTGAGTTAGAGGAA |
| NUP88 | Forward: AGTGCATTCAGTCCATCCTG  Reverse: GCCTTCAATGGTGTTCAGTTC | MYBBP1a | 1. CTCTCTTTGCAAACCTGTTTG  2. GAGACGAAGAAGCGCAAGAAA |
| WRAP53 | Forward: CTTGCGAATTTATAACCTGCCC  Reverse: GAGGACATCAGAGAATACCAGC | GEMIN4 | 1. GCAACCGTGTATCTGGACAAA  2. CAGCCTGGACTTTACAGTTAT |
| DHX33 | Forward: TCGCTGAAACCTCCATAACC  Reverse: GCTAACACCTCAAGACCACTG | DHX33 | 1. GTTGACACGGGCATGGTTAAA  2. GCTATCGCAAAGTGATCATTT |
| TSR1 | Forward: GAATTAAACCCCAAAAGGACCC  Reverse: GATCTGGGATAACCTCTGCTTG | WRAP53 | 1. GTTCCTGCATCTTGACCAATA  2. CACCAATCAGCGCATCTACTT |
| C1QBP | Forward: TTGATGTCAGTTCAGGCTCC  Reverse: TTGATGTCAGTTCAGGCTCC | C1QBP | 1. GCACCAGGAGTACATTACTTT  2. TCTGAATGGAAGGATACTAAT |
| GAPDH | Forward: ACATCGCTCAGACACCATG  Reverse: TGTAGTTGAGGTCAATGAAGGG | TSR1 | 1. TCAAGATTATGCTCGAATATT  2. TGATATGGAAGAAGGTCTTAA |

Table S2. The signature module for each drug derived by WGCNA analysis. (separate file)

Table S3. Pearson correlation analysis between basal gene expression and drug sensitivity of PARP inhibitors and cisplatin. (separate file)

Table S5. The prediction on response of ovarian cancer cell lines to PARP inhibitors and cisplatin. (separate file)

Table S6. The prediction on response of breast cancer cell lines to PARP inhibitors and cisplatin. (separate file)

Table S9. The prediction on response of a panel of primary breast cancer PDTX-derived tumor cells (PDTCs) to PARP inhibitor. (separate file)

Table S10. The prediction on response of a panel of primary ovarian cancer PDTCs to PARP inhibitor. (separate file)

Table S11. The prediction on response of patients with platinum-sensitive relapsed ovarian cancer treated with PARP inhibitor maintenance monotherapy. (separate file)
